# Human blood plasma biomarkers of diet and weight loss among centrally obese subjects in a New Nordic Diet intervention

**DOI:** 10.3389/fnut.2023.1198531

**Published:** 2023-06-15

**Authors:** Alessia Trimigno, Bekzod Khakimov, Morten Arendt Rasmussen, Lars Ove Dragsted, Thomas Meinert Larsen, Arne Astrup, Søren Balling Engelsen

**Affiliations:** ^1^Department of Food Science, Faculty of Science, University of Copenhagen, Frederiksberg, Denmark; ^2^COPSAC (Copenhagen Prospective Studies on Asthma in Childhood), Herlev and Gentofte Hospital, University of Copenhagen, Copenhagen, Denmark; ^3^Department of Nutrition Exercise and Sports, Faculty of Science, University of Copenhagen, Copenhagen, Denmark

**Keywords:** plasma metabolomics, ^1^H NMR, ketone bodies, weight loss, lipoproteins

## Abstract

**Scope:**

The New Nordic Diet (NND) has been shown to promote weight loss and lower blood pressure amongst obese people. This study investigates blood plasma metabolite and lipoprotein biomarkers differentiating subjects who followed Average Danish Diet (ADD) or NND. The study also evaluates how the individual response to the diet is reflected in the metabolic differences between NND subjects who lost or maintained their pre-intervention weight.

**Methods:**

Centrally obese Danes (BMI >25) followed NND (90 subjects) or ADD (56 subjects) for 6 months. Fasting blood plasma samples, collected at three time-points during the intervention, were screened for metabolites and lipoproteins (LPs) using proton nuclear magnetic resonance spectroscopy. In total, 154 metabolites and 65 lipoproteins were analysed.

**Results:**

The NND showed a relatively small but significant effect on the plasma metabolome and lipoprotein profiles, with explained variations ranging from 0.6% for lipoproteins to 4.8% for metabolites. A total of 38 metabolites and 11 lipoproteins were found to be affected by the NND. The primary biomarkers differentiating the two diets were found to be HDL-1 cholesterol, apolipoprotein A1, phospholipids, and ketone bodies (3-hydroxybutyric acid, acetone, and acetoacetic acid). The increased levels of ketone bodies detected in the NND group inversely associated with the decrease in diastolic blood pressure of the NND subjects. The study also showed that body weight loss among the NND subjects was weakly associated with plasma levels of citrate.

**Conclusion:**

The main plasma metabolites associated with NND were acetate, methanol and 3-hydroxybutyrate. The metabolic changes associated with the NND-driven weight loss are mostly pronounced in energy and lipid metabolism.

## Introduction

Human blood plasma contains hundreds of molecules related to metabolism (metabolites) as well as a diversity of lipoproteins, small particles involved in the transport of fats and cholesterol in the aqueous blood streams. Despite a strong push towards homeostasis, the levels of metabolites and lipoproteins in blood change with time depending on multiple factors such as diet, age, health, etc. The importance of diet on human health is well known, but the underlying molecular mechanisms remain elusive. For this reason, blood plasma has been increasingly employed in metabolomics studies to better understand the impact of diet on human metabolism and health (1).

In 2004, the new Nordic cuisine was developed by Nordic chefs and promoted as a sustainable, seasonal, and healthy diet. Soon after, in Denmark, the OPUS (optimal well-being, development and health for Danish children through a healthy New Nordic Diet) project was launched (clinical trial NCT01196610 https://clinicaltrials.gov/ct2/show/NCT01195610) (2). The project had an aim to develop a healthy New Nordic Diet (NND) based on regional production and growth, which could appeal to the public by its taste, healthiness and sustainability, and a low carbon footprint. The NND is characterized by a higher content of organic foods, including whole grains, nuts, berries, fruit and vegetables, fish and seafood, and a lower content of meat. This led to designing a SHOPUS (shop in OPUS) study for better understanding the impact of NND on centrally obese individuals over a period of 6 months. The Average Danish Diet (ADD) was used as a control. The recruited subjects could freely choose among products from the assigned diet in a special shop, set up at the University of Copenhagen. Subjects who followed NND displayed a greater weight loss, a larger decrease in blood pressure, a small non-significant drop in LDL cholesterol, and a lower frequency of the metabolic syndrome, when compared to ADD subjects (2, 3). During the intervention, urine and blood samples were collected at three time points, 0, 12 and 26 weeks, and analyzed for metabolites. Previous studies using mass spectrometry (MS) have shown differences in the plasma (4, 5), and urine metabolomes (6) between subjects who followed NND or ADD. These studies have shown that the NND induces metabolic changes in the blood, mainly related to the higher intakes of whole grain, vegetables, and fish (4, 5). It was also found that plasma concentrations of gut derived metabolites such as vaccenic acid and 3-hydroxybutyric acid were higher in NND subjects who lost more body weight, while lactic acid levels were found to be higher amongst NND subjects who maintained their body weight after intervention (5).

This study investigates the human blood plasma metabolites using ^1^H NMR spectroscopy. The major advantages of NMR are that it is unbiased and inherently quantitative toward a broad range of metabolite classes present in human blood plasma (7–10), and that the same NMR analysis can be exploited for robust quantification of the plasma lipoprotein profile (7, 11). The aim of the present study is twofold; (1) to identify differences in plasma metabolites and/or lipoprotein patterns related to the diet (NND versus ADD) and (2) to investigate if metabolic changes related to NND driven weight loss are associated with specific biomarkers and if these are related the dietary intervention. This could provide additional insights into underlying mechanisms behind diet-induced weight loss.

## Experimental section

### Study design

During a 6 month period between October 2010 and July 2011, a randomized, parallel, and controlled dietary intervention was conducted to investigate the impact of the NND on centrally obese Danes (waist circumferences ≥80 cm for women and ≥94 cm for men). The number of recruited subjects was 181 (53 males, 128 females), between 20 and 66 years old (average: 42 years old). Moreover, the participants had one or more of the following characteristics: impaired fasting glucose level >5.6 mmol/L, plasma triglyceride concentrations ≥1.7 mmol/L, HDL-cholesterol concentrations ≤1.03 mmol/L for men and ≤1.29 mmol/L for women, and systolic/diastolic blood pressure >130/85 mm Hg. Exclusion criteria included: diagnosed diabetes (both type 1 and 2), total cholesterol ≥9 mmol/L, triglyceride concentration ≥3 mmol/L, familial hypercholesterolemia, food allergies contrasting with the intervention, pregnancy, or lactation. In addition, subjects who lost >2 kg in the preceding 2 months were excluded.

The control to the NND was ADD, and the subjects were randomly divided between the two diets in a 3:2 ratio using simple block randomization, with a stratification done according to age (<45 or ≥ 45 years), BMI (<33 or ≥ 33 kg/m^2^), and whether they were enrolled as individuals or couples. The two diets differed in the composition of 15 food groups that were specific for NND, and in macronutrients, including total intake of proteins, carbohydrates, and fats. NND was characterized by a higher fish, whole grain, fruit, and vegetable consumption and by lower intakes of terrestrial meat, compared to ADD. A detailed description of the two diets is given in a previous article (2).

**Figure 1:**
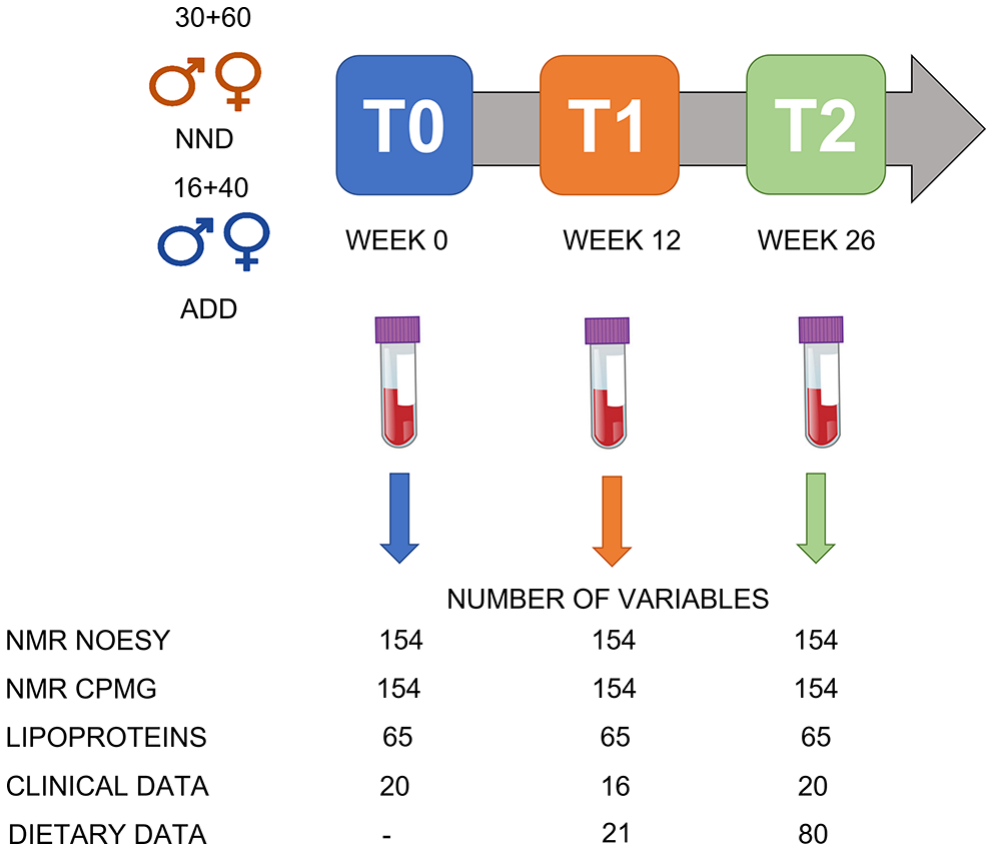
An overview of the study design. A total of 146 subject were included: 90 in the NND group (60 females and 30 males) and 56 in the ADD group (40 females and 16 males). Samples were collected at T0 (week 0), T1 (after 12 weeks) and T2 (after 26 weeks of intervention). A total of 438 plasma samples were collected over the three time points. The NMR spectra (NOESY and CPMG; see under data acquisition) were converted into a metabolite table including 65 SS, 33 SUS, and 56 BINS after processing the spectral datasets in SigMa. The lipoprotein data include the absolute concentrations (mg/dL) of 65 lipoproteins predicted from the NOESY spectra. The clinical data include different anthropometric and clinical parameters variables collected during the study, including bodyweight, height, age, and blood parameters. Dietary data included 21 variables from food diaries at T1 and T2, and additional 59 variables from shop data at T2.

An overview of the study design is given in Figure 1. Fasting blood samples and clinical parameters were collected at T0 (week 0, at the beginning of the trial), T1 (week 12), and T2 (week 26). The measured parameters were body weight, waist and hip circumference, sagittal diameter, blood pressure, and body composition, measured by dual-energy X-ray absorptiometry (Lunar Radiation Co). For more details see the work by Poulsen and colleagues (2). The EDTA (ethylenediaminetetraacetic acid treated) blood plasma samples were prepared and stored at −80°C until NMR analysis at the University of Copenhagen. A total of 146 subjects completed the intervention study including 90 NND (60 females, 30 males) and 56 ADD (40 females and 16 males) which resulted in a total of 438 plasma samples collected over the three time points.

The SHOPUS study has been approved by the Regional Ethics Committee of Greater Copenhagen and Frederiksberg (H-3-2010-058) and by the Danish Data Protection Agency (2007-54-0269).

### Sample preparation and ^1^H NMR data acquisition

Plasma samples were thawed on ice for 60 min. A 300 μL plasma aliquot was then mixed with 300 μL phosphate buffer (pH 7.4), containing 0.8 mg mL^−1^ trimethylsilylpropionate (TSP) in 2 mL Eppendorf tubes (Eppendorf, Hamburg, Germany), and then transferred to 5 mm NMR tubes (Bruker Biospin Gmbh, Rheinstetten, Germany) (9). NMR analysis was performed on a Bruker Avance III 600 spectrometer (Bruker Biospin Gmbh, Rheinstetten, Germany) operating at a Larmor frequency of 600.13 MHz for protons, equipped with a double tuned cryo-probe (TCI) set for 5 mm sample tubes and a cooled autosampler (SampleJet). One-dimensional proton nuclear magnetic resonance (^1^H NMR) spectra were acquired from all plasma samples using both the Carr–Purcell Meiboom–Gill (CPMG) experiment and the NOESY-presat pulse sequences from Bruker’s library. The former suppresses, apart from water resonance, also resonances from large molecules (i.e., proteins), and can thus better facilitate the identification of small molecules. The latter, instead, only suppresses water resonance, providing a more unbiased quantitative picture of the sample composition. All experiments were performed at 310 K with a fixed receiver gain (RG) of 40.3. A total of 64 scans were acquired and the measured free induction decays (FID) were collected into 128k data points. The automation program controlling sample measurements included the acquisition routines for locking, automatic tuning and matching, shimming, pulse calibration, and optimized pre-saturation power for each sample, as well as automatic data processing including Fourier transformation (FT) of FID, with a Lorentzian line-broadening of 0.3 Hz before FT, phasing, and baseline correction.

### ^1^H NMR data processing

**Figure 2:**
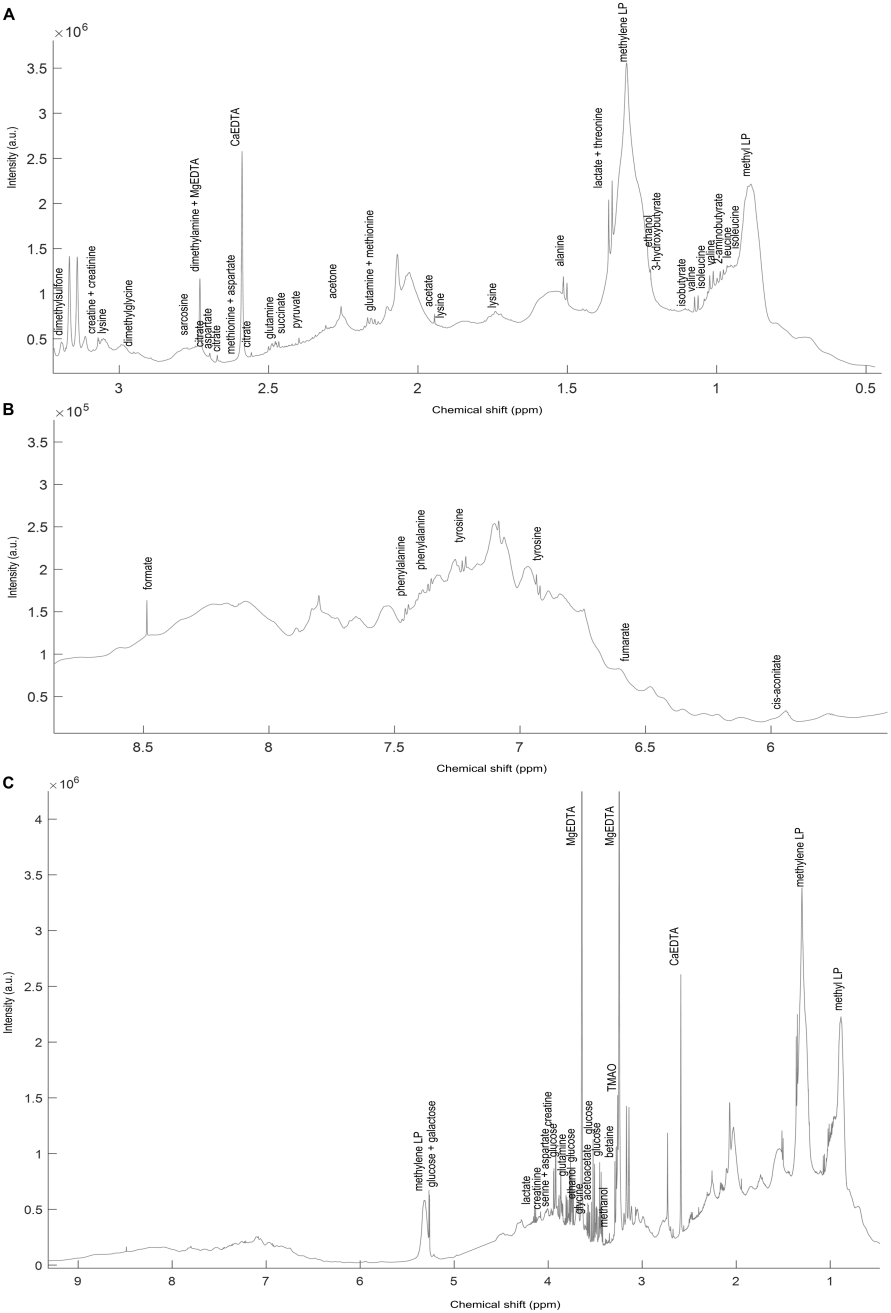
A representative spectrum **(C)** of the ^1^H NMR spectra (NOESY) acquired on the NND subjects. Annotated signals represent some of SigMa based quantified blood plasma metabolites. The **(A)** aliphatic (3.1–0.8 ppm) and **(B)** aromatic (8.5–6 ppm) regions are also represented zoomed-in. The full list of annotated metabolites, with relative signal chemical shift (ppm) and multiplicity is reported in Supplementary Table S5.

Raw ^1^H NMR spectra were converted to a metabolite concentration table using the SigMa software (12). The SigMa based processing included reference alignment (towards the TSP signal at 0.0 ppm) and pre-alignment of larger spectral regions using the *icoshift* method (13), followed by interval recognition where the entire spectra are divided into smaller regions of signature signals (SS) of known human blood metabolites, signals of unknown spin systems (SUS), and BINS representing complex regions containing unresolved signals of more than one metabolite. After interval recognition, SigMa quantified SS and SUS variables using a one-component multivariate curve resolution (MCR) based modelling (14). BINS are instead quantified using an integration approach, summing all data points of a given interval for each sample. As a unique feature, ^1^H NMR spectra of plasma also contain information about the blood lipoproteins (7). Extraction of this information typically relies on prediction models based on partial least squares regression of the spectral region, 1.4–0.6 ppm, to reference lipoprotein values stemming from ultracentrifugation (9). In this work a lipoprotein dataset consisting of absolute concentrations of 65 LPs was generated from the NOESY 1H NMR spectra using previously described lipoprotein prediction models (9, 15) (Figure 2). The variables include concentrations of the main lipoprotein classes (VLDL, IDL, LDL, HDL), and subclasses of these (LDL-1 to LDL-6, and HDL-1 to HDL-3, given in increasing density and decreasing size). For each subclass, cholesterol, free cholesterol, triglycerides, phospholipids, and apolipoproteins A1 and B were quantified. More detailed information on the LPs is reported in Supplementary Table S1.

### Data analysis

**Figure 3:**
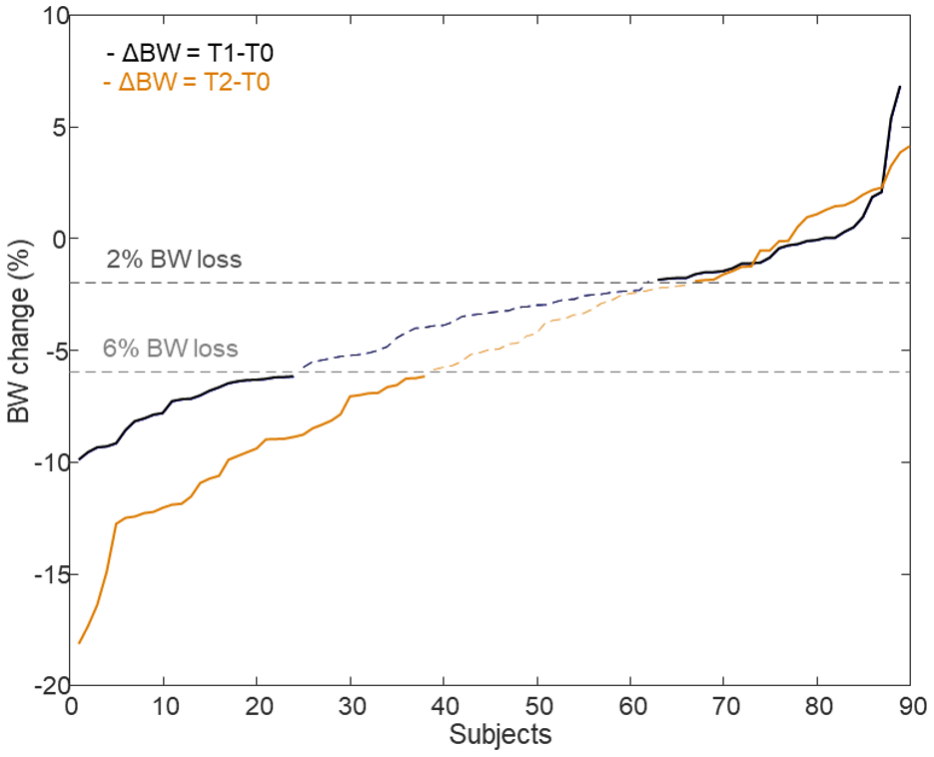
Delta body weight of subjects who were assigned to the NND diet. A total of 62 subjects were selected as BW losers (>6%), 24 from T1 time point (ΔBW = T1–T0) and 38 from T2 time point (ΔBW = T2–T0) (22 in common between the two time points). Likewise, 52 individuals, 28 at T1 and 24 at T2 (19 in common), were identified as BW maintainers (<2%) or even gained weight.

The proton NMR spectral ensembles (NOESY and CPMG spectra) were firstly resolved into metabolite tables and LP tables for the different time points as described above. The datasets representing **X**_T1_ (T1; 12 weeks) and **X**_T2_ (T2; 26 weeks) were further corrected for baseline (T0 time values) as follows: **ΔX**_T1_ = **X**_T1_–**X**_T0_ and **ΔX**_T2_ = **X**_T2_–**X**_T0_. This procedure yielded a total of six datasets, **ΔX**_T1_NOESY,_ **ΔX**_T1_CPMG,_ **ΔX**_T1_LP,_ **ΔX**_T2_NOESY,_ **ΔX**_T2_CPMG,_ and **ΔX**_T2_LP_ to be scrutinized for evaluating metabolic differences related to the diet, NND vs. ADD, and metabolic changes related to weight loss of the NND subjects. The weight loss factor was defined as described before (5), by stratifying the NND subjects into two groups: *weight losers* (*n* = 62) with at least 6% weight loss according to their body weight at inclusion and *weight maintainers* (*n* = 52) who lost ≤2% of their pre-intervention body weight. ADD subjects showed very limited weight change, and thus were excluded from this investigation (2). For NND participants, consistent *weight losers* (>6%) and consistent *weight maintainers* (<2%) both at T1 and T2 were limited to only 22 and 19 subjects, respectively (Figure 3). Additional data used for stratification of subjects included dietary recordings, anthropometric and clinical parameters which have been published elsewhere (2). All datasets were mean centered and scaled to unit standard deviation prior to multivariate data analysis.

Principal component analysis (PCA) (16) was used to explore the datasets, for outlier detection as well as to investigate variations related to diet and weight loss. ANOVA-simultaneous component analysis (ASCA) (17) with permutation testing (*n*_perm._ = 1,000) was applied to quantify variations explained by the study design factors, diet (NND vs. ADD) and weight loss (NND weight losers vs. NND weight maintainers). Partial least squares-discriminant analysis (PLS-DA) (18) was employed to identify metabolite patterns that discriminate between the two diet groups or weight losers vs. weight maintainers. Variable selection (variables selected 70% of the times by the model) and validation of the PLS-DA models were performed as previously described (5). One-way ANOVA using false discovery rate (FDR, 5%) correction was employed to identify individual metabolites or lipoproteins that were different between the two diets or between the weight losers and weight maintainers. Data analysis was performed in MATLAB R2015b (The Mathworks Inc., Natick, MA) using customized scripts written by the authors and additionally using PLS Toolbox 8.7.1 (Eigenvector Research, Manson, United States). Scripts and data matrices are available upon request.

## Results

### The SHOPUS cohort

The averages and standard deviations of the main parameters, relevant to this study, of the individuals following the two diets, NND and ADD, are presented in Table 1. The NND group lost more weight, reduced their diastolic (DBP), systolic (SBP) blood pressure, and homeostatic model assessment for insulin resistance (HOMA-IR) factor during the intervention. The reduction in blood pressure was partially related to the greater weight loss in NND, but also to the specific diet pattern of NND (2). Further information on the effect of NND, compared to ADD, on subjects’ clinical parameters including fat mass, waist and hip circumference, fasting insulin and glucose, and CRP can be found in Poulsen et al. (2).

**Table 1:** An overview of the two dietary groups, New Nordic Diet (NND), and Average Danish Diet (ADD).

	NND	ADD
Age (y)^a^	44.15 ± 13.1	40.8 ± 13.2
Males (*n*)	30	16
Females (*n*)	60	40
Starting BW at T0 (kg)^a^	91.2 ± 16.2	90.1 ± 19.3
ΔBW at T1 (kg)^b^	−3.24 ± 0.31	−1.48 ± 0.29
ΔBW at T2 (kg)^b^	−4.77 ± 0.48	−1.46 ± 0.44
ΔHOMA-IR at T2^b^	−3.08 ± 0.13	0.10 ± 0.11
ΔDBP at T2 (mm Hg)^b^	−0.52 ± 0.79	−0.08 ± 0.92

a Mean ± SD (standard deviation).

b Mean ± SEM (standard error of the mean).

### Description of the NMR data

A representative ^1^H NMR spectrum of human blood plasma is shown in Figure 2. The spectrum is largely dominated by ^1^H resonances of methyl and methylene groups corresponding to fatty acids and lipoproteins (1.4–0.6 ppm). Overall, the most abundant plasma metabolites included cholesterol-C18 (CH_3_, *δ* 0.70, s), lactic acid (CH_3_, *δ* 1.35, d, J 6.93 Hz; CH, *δ* 4.14, q, J 6.93 Hz), alanine (CH_3_ *δ* 1.49, d, J 7.14 Hz), valine (CH_3_ *δ* 0.98, d, CH_3_ *δ* 1.02, d, J 7.07 Hz) and glucose (CH *δ* 3.20–4.00, m, CH *δ* 5.27, d, 3.8 Hz). Using Signature Mapping (SigMa) software the ^1^H NMR data was converted into a metabolite table. The resulting table consisted of 154 variables: 65 were SS of known plasma metabolites, 33 SUS and 56 BINS (Supplementary Table S5). Both CPMG and NOESY spectra were processed in the same way, and both contained 154 metabolite variables. Furthermore, the same NMR data (only NOESY) were used for prediction of the lipoprotein profiles which generated two additional data sets, **ΔX**_T1_LP_ and **ΔX**_T2_LP_, consisting of absolute concentrations of 65 lipoproteins (9) (Supplementary Table S1).

### Diet related metabolic differences in blood plasma (NND vs. ADD)

**Figure 4:**
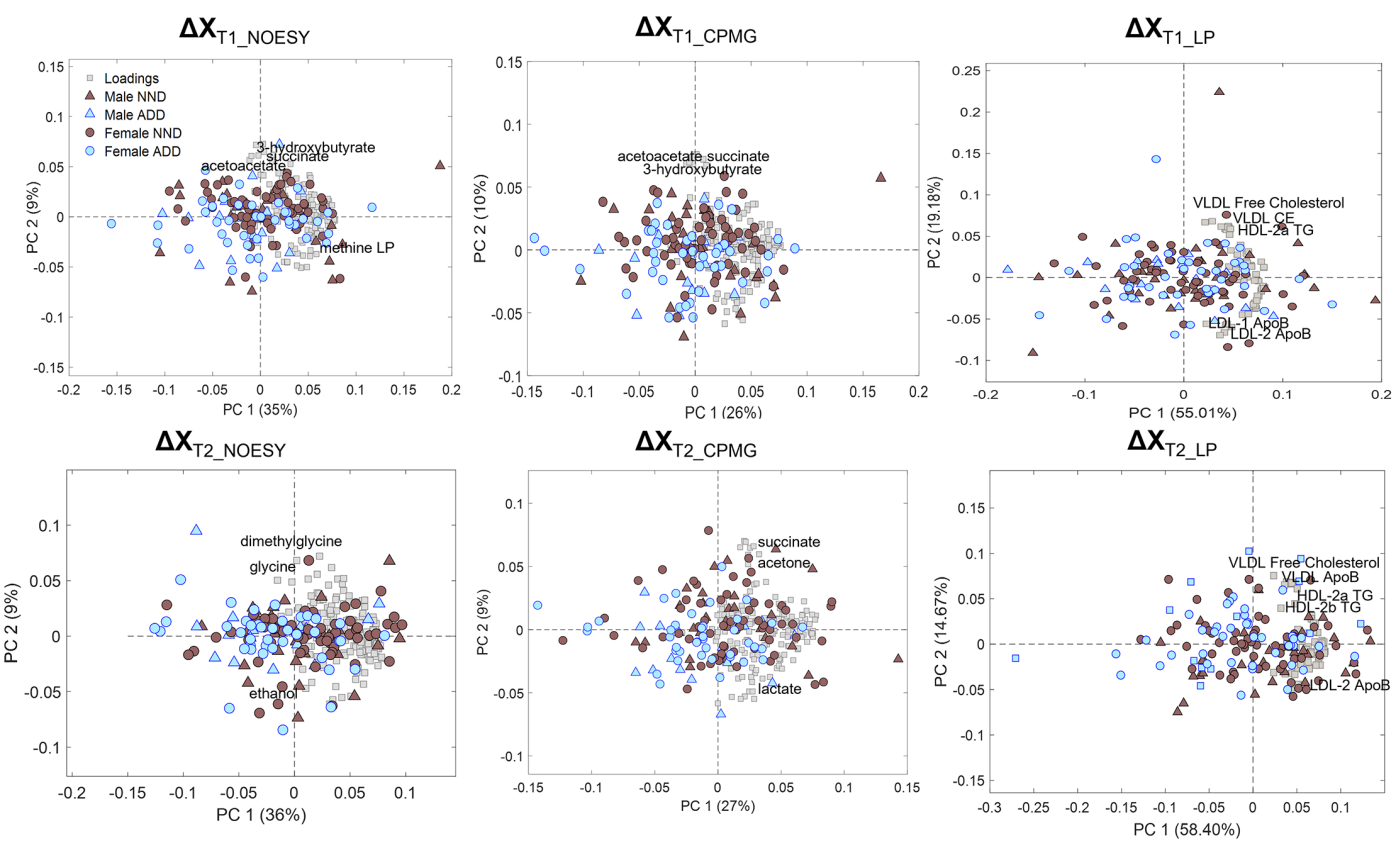
PCA biplots of the six matrices used for data analysis: ΔX_T1_NOESY_, ΔX_T1_CPMG_, ΔX_T1_LP_, ΔX_T2_NOESY_, ΔX_T2_CPMG_, and ΔX_T2_LP_, colored by diet class (NND in brown, ADD in light blue) and represented with either triangles (males) or circles (females) according to sex. Loadings are represented by grey squares, and the most relevant are annotated.

**Figure 5:**
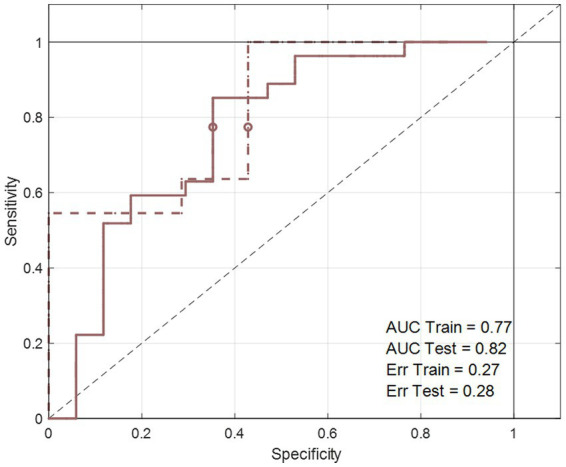
The area under the curve of the receiver operating characteristics (AUCROC) obtained from the PLS-DA model developed for BW loss effect. These results represent the final PLS-DA models after variable selection and were developed on **ΔX**_T2_NOESY_NND_BW_, using a training set of 44 subjects and tested on 18 subjects.

Principal component analysis of the metabolite and lipoprotein datasets revealed a weak pattern related to the diet (Figure 4). This was subsequently confirmed by ASCA which revealed that all datasets at both T1 and T2 (except ΔX_T1_LP_) showed a significant effect of diet with explained variations ranging from 0.6 to 4.8% (Supplementary Table S6). The NOESY dataset at T2 (**ΔX**_T2_NOESY_) showed the largest fraction (4.8%) of the variation explained by diet (*p* < 0.0001). Further investigation of the effect of the diet was performed using PLS-DA which allowed to find the pattern of metabolites discriminating NDD and ADD subjects using above mentioned datasets. Classification performances of the PLS-DA models developed on the six datasets after variable selection are summarized in Table 2. The best performing PLS-DA model was developed on ΔX_T1_NOESY_ dataset (AUC = 0.80, error = 28%) and included 75 variables to be important markers of the diet, the second best was developed on ΔX_T2_NOESY_ dataset (AUC = 0.74, error = 28%, 115 variables used). The consistently selected 59 variables between these two models included 22 plasma metabolites (see Table 3) such as amino acids (lysine, phenylalanine, tyrosine, serine, asparagine), organic acids (acetic acid, lactic acid, citric acid, fumaric acid, succinic acid), and ketone bodies (acetone, acetoacetate and 3-hydroxybutyrate) (see Figure 5).

**Table 2:** Results from PLS-DA after variable selection.

Matrix	*N*. variables used	N. LVs	Prediction AUC	Prediction error	Training AUC (CV)	Training error (CV)
ΔX_T1_NOESY_	75	4	0.80	0.28	0.90	0.18
ΔX_T2_NOESY_	115	2	0.74	0.28	0.83	0.27
ΔX_T1_CPMG_	55	2	0.74	0.33	0.84	0.17
ΔX_T2_CPMG_	89	3	0.70	0.38	0.87	0.18
ΔX_T1_LP_	32	1	0.51	0.55	0.37	0.57
ΔX_T2_LP_	38	4	0.64	0.41	0.75	0.32

The number of variables and latent variables used is reported for each matrix, together with the AUC and error values for the training set (88 subjects) and prediction sets (58 subjects). Cross-validation using venetian blinds, with 8 data splits and one sample per blind, was used.

**Table 3:** A list of metabolite and lipoprotein variables found to be associated with the diet effect by both PLS-DA and one-way ANOVA.

Variable name	Class	*p*-value	Effect size	Median NND % variation	Median ADD % variation	Matrix
Methanol	OH	6.56E−04	12.43	1.06	−1.60	ΔX_T1_NOESY_
3-hydroxybutyrate	KB	1.83E−03	8.62	1.34	−0.67	ΔX_T2_NOESY_
Acetate	OA	6.62E−07	21.17	1.06	−1.83	ΔX_T2_NOESY_
Acetoacetate	KB	5.41E−03	6.59	0.87	−1.67	ΔX_T2_NOESY_
Betaine	AA	3.31E−03	7.34	6.78	−2.94	ΔX_T2_NOESY_
Cis-aconitate	OA	3.68E−04	11.22	0.51	−0.92	ΔX_T2_NOESY_
Citrate	AA	1.01E−02	5.59	4.55	−4.29	ΔX_T2_NOESY_
Creatine	AA	4.41E−02	3.59	1.09	−0.89	ΔX_T2_NOESY_
Dimethylamine	ONC	1.16E−03	9.35	4.29	−3.85	ΔX_T2_NOESY_
Fumarate	OA	2.80E−04	11.62	1.54	−2.09	ΔX_T2_NOESY_
Galactose	SG	1.94E−03	8.46	3.42	−3.58	ΔX_T2_NOESY_
Glucose	SG	3.00E−03	7.51	3.32	−3.79	ΔX_T2_NOESY_
Glutamine	AA	8.67E−03	5.85	5.16	−2.03	ΔX_T2_NOESY_
Leucine	AA	1.93E−02	4.70	0.22	−0.51	ΔX_T2_NOESY_
Lysine	AA	6.96E−04	10.13	4.73	−4.76	ΔX_T2_NOESY_
Methanol	OH	6.62E−07	20.62	0.86	−1.59	ΔX_T2_NOESY_
Methine LP	LP	2.49E−02	4.35	6.17	−1.02	ΔX_T2_NOESY_
Methyl LP	LP	3.38E−03	7.28	5.22	−4.61	ΔX_T2_NOESY_
Phenylalanine	AA	2.23E−02	4.50	3.16	−1.74	ΔX_T2_NOESY_
Succinate	OA	3.00E−03	7.51	1.11	−2.11	ΔX_T2_NOESY_
TMAO	ONC	1.75E−03	8.76	4.97	−3.44	ΔX_T2_NOESY_
Acetone	KB	3.64E−02	7.33	21.38	−10.94	ΔX_T1_CPMG_
Glutamine	AA	4.52E−02	6.81	20.26	19.94	ΔX_T1_CPMG_
3-hydroxybutyrate	KB	2.93E−04	13.45	1.31	−2.89	ΔX_T2_CPMG_
Acetate	OA	5.55E−07	21.55	1.03	−1.82	ΔX_T2_CPMG_
Acetoacetate	KB	6.89E−03	6.66	0.97	−1.58	ΔX_T2_CPMG_
Acetone	KB	1.45E−02	5.49	12.79	−9.39	ΔX_T2_CPMG_
Betaine	AA	2.08E−02	4.99	8.17	−0.04	ΔX_T2_CPMG_
Cis-aconitate	OA	3.69E−03	8.23	0.45	−1.06	ΔX_T2_CPMG_
Citrate	OA	2.33E−02	4.83	4.77	−2.36	ΔX_T2_CPMG_
Creatine	AA	3.96E−02	4.11	1.47	−1.02	ΔX_T2_CPMG_
Dimethylamine	ONC	3.69E−03	8.31	4.32	−3.48	ΔX_T2_CPMG_
Ethanol	OH	6.74E−03	6.79	0.30	−0.42	ΔX_T2_CPMG_
Galactose	SG	3.69E−03	8.22	3.32	−2.54	ΔX_T2_CPMG_
Glucose	SG	3.69E−03	8.10	4.01	−3.42	ΔX_T2_CPMG_
Glutamine	AA	1.02E−02	6.11	6.59	−1.56	ΔX_T2_CPMG_
Methanol	OH	4.49E−03	7.69	1.41	−1.35	ΔX_T2_CPMG_
Methine LP	LP	4.42E−02	3.96	7.06	−0.07	ΔX_T2_CPMG_
Succinate	OA	6.01E−04	11.25	0.89	−3.27	ΔX_T2_CPMG_
TMAO	ONC	2.75E−03	9.10	4.34	−4.21	ΔX_T2_CPMG_
Main Fraction Cholesterol	LP	8.50E−03	5.06	5.60	−2.77	ΔX_T2_LP_
Sub Fraction Cholesterol	LP	6.90E−03	5.64	5.68	−3.17	ΔX_T2_LP_
HDL Cholesterol	LP	3.10E−03	9.30	5.73	−5.57	ΔX_T2_LP_
HDL-2b Cholesterol	LP	8.00E−04	14.28	14.78	−10.07	ΔX_T2_LP_
HDL-2b Free Cholesterol	LP	2.30E−03	10.08	10.76	−4.54	ΔX_T2_LP_
Main Fraction Phospholipids	LP	6.20E−03	5.85	8.82	−4.41	ΔX_T2_LP_
HDL-2b Phospholipids	LP	4.60E−03	7.67	13.08	−10.76	ΔX_T2_LP_
Plasma ApoA1	LP	9.20E−03	5.03	3.00	−1.81	ΔX_T2_LP_
Main Fraction ApoA1	LP	1.08E−02	4.80	4.10	−0.28	ΔX_T2_LP_
Sub Fraction ApoA1	LP	5.40E−03	6.11	4.01	−2.59	ΔX_T2_LP_

Median variance is calculated as the median % of the individual variance from T0, for example for T1 data: median ((T1–T0/T0)*100). For metabolites displaying more than one significant signal, only one is shown. Representative SUS BINS are listed in Supplementary Table S7. AA, amino acid (and derivatives); KB, ketone body; LP, lipoprotein; OA, organic acid; OH, alcohol; ONC, organic nitrogen compounds; SG, sugar.

To test the hypothesis that plasma metabolites and lipoproteins differ in their concentrations between the two different diet groups, one-way ANOVA was employed. Most of the metabolites and LPs found to be associated with diet from the PLS-DA based variable selection were different between NND versus ADD in ANOVA (Table 3). All the 11 LPs which were significant for the diet effect, were found at higher levels in the NND subjects. The HDL-2b cholesterol showed the largest diet effect and were found at higher concentrations in blood of NND subjects (15% increase in NND vs. 10% decrease in ADD at T2) compared to ADD subjects. A total of 137 metabolite variables were found to be different between NND vs. ADD in one-way ANOVA when including all datasets. These metabolite variables included ketone bodies (acetone, acetoacetate, and 3-hydroxybutyrate), glucose, methanol, TMAO, organic acids like citrate and succinate, and amino acids like betaine and glutamine. The biggest effect is observed for acetate (>20% at T2), followed by methanol (8%–21%), 3-hydroxybutyrate (13%), and fumaric acid (12%), all found at higher levels in NND subjects.

### Comparison of blood plasma lipoproteins and metabolites between weight losers and maintainers in the NND subjects

Initially, a PCA was carried out on the datasets corresponding to the baseline points, T0 time point (**X**_T0_NND_BW_ dataset—see Supplementary Table S4), and showed no trend associated with weight loss among NND subjects (data not shown). ASCA performed on the same datasets showed no effect of the weight loss at T0. Likewise, a PLS-DA model developed to differentiate between weight losers and weight gainers at T0 (**X**_T0_NND_BW_) failed to classify the two groups at T0 (examples for the **X**_T0_NOESY_NND_BW_ datasets shown in Supplementary Figure S1). Accordingly, no metabotypes related to weigh loss at T0 could be identified.

Baseline corrected T1 and T2 datasets, however, showed weak to moderate BW effect. In PCA, no real separation is observed between weight losers and maintainers (examples for the **ΔX**_T1_NOESY_NND_BW_ and **ΔX**_T2_NOESY_NND_BW_ datasets shown in Supplementary Figure S1). However, ASCA showed a significant weight loss effect on **Δ**T2 datasets, **ΔX**_T2_CPMG_NND_BW_ and **ΔX**_T2_NOESY_NND_BW_, (*p*-value = 0.02 and 0.04, respectively), with 3.2% and 3.4% of the variation in the data being associated with the weight loss, respectively. Interestingly, a significant weight loss effect was also observed in the **ΔX**_T1_LP_NND_BW_ dataset (*p*-value = 0.02 and 5.7% variation). The effect of BW loss was further investigated by PLS-DA (Table 4). The best result (AUC = 0.82, error = 28%) was obtained using **ΔX**_T2_NOESY_NND_BW_ (Figure 5). One signal from citrate and an unknown singlet at 7.89 ppm, were consistently found to be BW related in PLS-DA and ANOVA (*p* < 0.05 with FDR correction) performed to test BW effect (Table 5). Despite the fact that none of the LP variables shows differences between NND weight losers and maintainers, some LP, including total free cholesterol, apolipoprotein B subfractions and LDL cholesterol esters, were recurrently selected by PLS-DA models developed to classify these two types of NND subjects (Supplementary Table S8).

**Table 4:** Results from PLS-DA prediction of BW loss after variable selection.

Matrix	*N*. variables used	*N*. LVs	Prediction AUC	Prediction error	Training AUC (CV)	Training error (CV)
ΔX_T1_NOESY___NND_BW_	3	1	0.84	0.27	0.62	0.38
ΔX_T2_NOESY_NND_BW_	77	2	0.82	0.28	0.77	0.27
ΔX_T1_CPMG_NND_BW_	2	1	0.55	0.47	0.83	0.24
ΔX_T2_CPMG_NND_BW_	98	3	0.66	0.30	0.74	0.39
ΔX_T1_LP_NND_BW_	11	1	0.55	0.47	0.71	0.41
ΔX_T2_LP_NND_BW_	35	2	0.71	0.28	0.81	0.30

The number of variables and latent variables used is reported for each matrix, together with the area under the curve (AUC) and error values for the training set (37 subjects at T1, 44 at T2) and prediction sets (15 subjects at T1, 18 at T2). Cross-validation (CV) using venetian blinds, with 8 data splits and one sample per blind, was used.

**Table 5:** Metabolites and lipoproteins selected by PLS-DA variable selection (variables selected 70% of the times by the model) and significant in one-way ANOVA on discrimination of body weight (BW) loss calculated on the matrix reported.

Variable name	*p*-value	Effect size	Median % variation from T0 BW losers	Median % variation from T0 BW maintainers	Matrix
Citrate3	0.03	19.63	14.44	−3.79	ΔX_T2_NOESY_NND_BW_
SUS33_s^a^	0.03	18.53	1.43	−0.86	ΔX_T2_NOESY_NND_BW_
bin32	0.03	18.21	11.47	−4.54	ΔX_T2_NOESY_NND_BW_

a SUS, signature signal of unknown spin system; s, singlet.

### Associations between plasma metabolites and lipoproteins with anthropometric and clinical parameters in NND and ADD subjects

**Figure 6:**
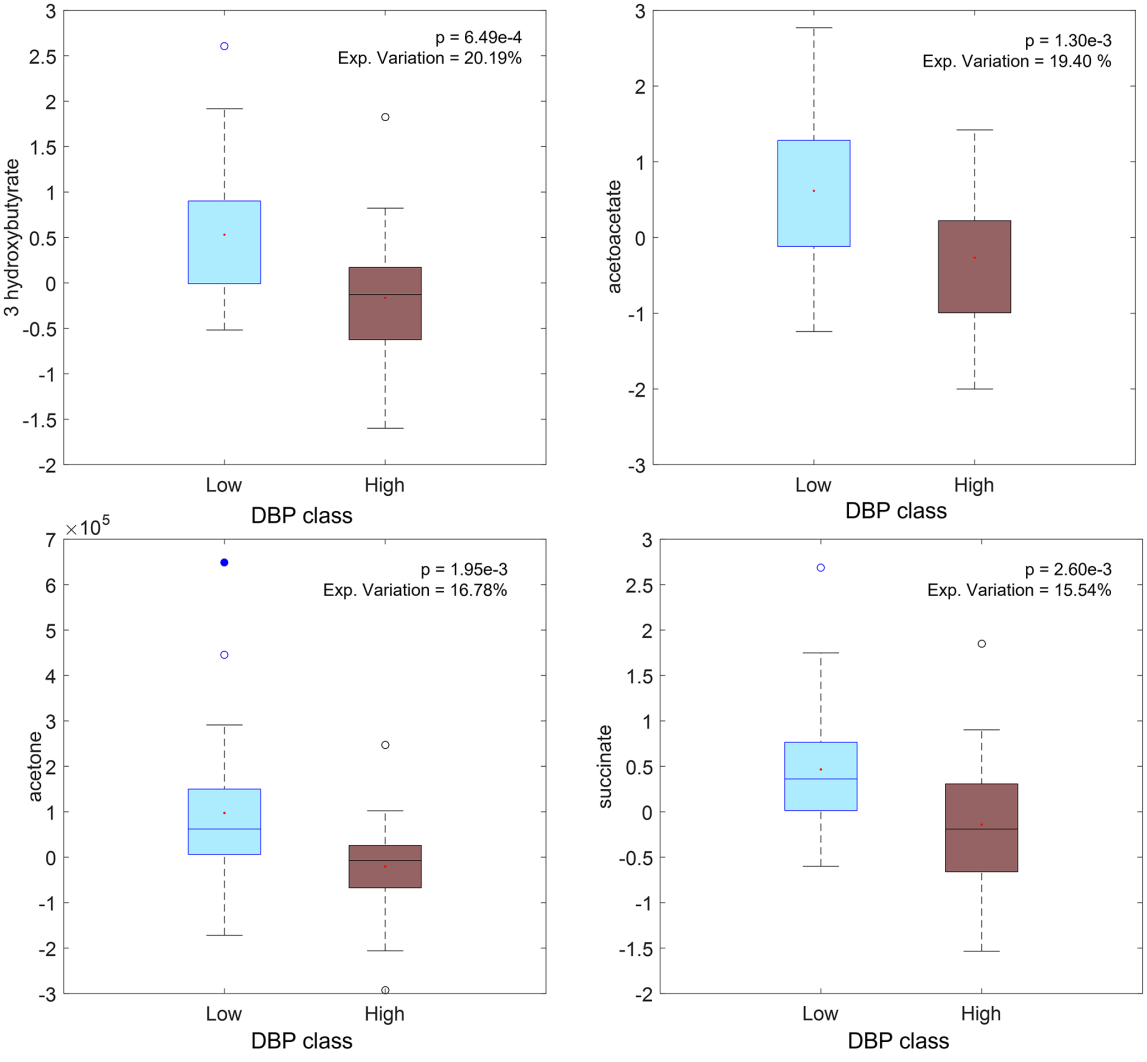
Boxplot of significant metabolites (from ΔX_T2_CPMG_NND_ matrix of selected subjects) related to diastolic pressure (DBP) changes: low values of ΔDBP on the left (DBP decreasing during intervention) and high values on the right (DBP increasing during intervention).

NND and ADD subjects were stratified separately according to their Δ anthropometric and clinical variables (e.g., ΔDBP_T2_ **=** DBP_T2_–DBP_T0_). One-way ANOVA was applied to evaluate differences in individual metabolite or lipoprotein levels between the two groups, low 1/3 versus high 1/3 tertiles. It was found that several metabolites, were inversely associated with the increase in DBP from baseline to T1 (increased by up to 53%), including acetoacetic acid, acetone and succinate, and T2 (increased up to 31%), including the former metabolites and another ketone body (3-hydroxybutyric acid), in the NND group (Supplementary Tables S9, S10). These metabolites were all found at higher levels for subjects for whom DBP decreased during the intervention (Figure 6). It is to be noted, though, that the signature signal of succinic acid was partially overlapping with one peak from 3-hydroxybutyrate, and thus, that could affect the result and the increase in this interval could be simply related to the ketone body increase in low DBP group.

## Discussion

### Metabotype and effect of diet

Clear metabolite patterns associated with the NND and ADD diets, respectively, were observed at T1 and T2. Metabolites from different chemical classes and from diverse parts of the metabolism were found to be part of the pattern distinguishing NND from ADD, and all these metabolite markers appeared at higher levels in the NND group. Few of them were directly related to the diet, with the possible exception of ethanol and TMAO, which may signify a higher ethanol and fish intake in NND. Ketone bodies recurrently showed up as markers of NND. These metabolites can arise from fatty acid metabolism. The increased energy % of PUFAs in NND (2) could give rise to this increase in ketone bodies (19, 20). The reduced energy intake in NND could also explain the higher levels of ketone bodies, together with an increase in TCA cycle intermediates (i.e., citrate), glucose and acetate, which previously has been correlated to a higher fat metabolism (21) improved insulin regulation (22), and hepatic gluconeogenesis (23). The increase of ketone body metabolism has recently been proposed as beneficial in the long term, as it starts an adaptive response by activating cell-protective mechanisms, up-regulating anti-inflammatory and anti-oxidative activities, and improving mitochondrial function and growth (23).

Glutamine, ethanol and methanol were also amongst the recurrent NND markers. Glutamine has previously been related to whole grain diets (24). The level of methanol in the blood was found to be higher in NND subjects, while the opposite has been reported from urine samples (6). Endogenous methanol can stem from gut microbiota fermentation (25–27) or by transformation from S-adenosyl methionine (SAM) (27). Methanol has previously been related to the intake of fresh fruit and vegetables, juices and fermented beverages (6) and inversely related to high fat diet (28). Plasma ethanol, which may originate from alcohol intake, can also be formed endogenously or by the microbiota from acetaldehyde, which in turn can be generated from various precursors such as pyruvate or alanine (29). Due to the randomization in SHOPUS an increase in alcohol intake in the NND group is less likely than a change in its microbial or endogenous formation.

**Figure 7:**
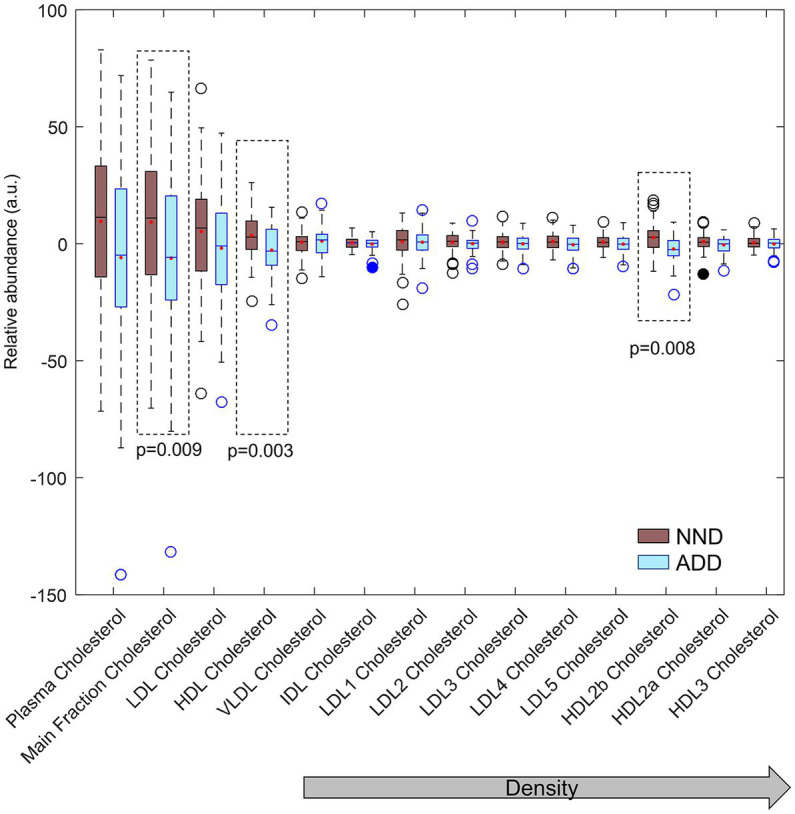
Total, main fraction, VLDL, IDL, LDL and HDL cholesterol fractions and subfractions compared between the two diet groups (NND in brown and ADD in light blue) at T2. Cholesterol main fractions HDL, and HDL-2b (inside dotted lines) differed between the two diet groups by ANOVA.

Limited significant differences were also observed in lipoprotein levels between ADD and NND diets. The level of cholesterol in apolipoprotein A1 sub-fractions, and in particularly in HDL-2b sub-fraction, was found to be higher in NND subjects (Figure 7) as opposed to previously published total cholesterol level in blood (2). In general, there seem to be small lipid-lowering effects in the NND group, but effect sizes are very small.

The changes in the LPs can be traced back to differences in macronutrient intakes of the two diets. The NND includes higher intakes of carbohydrates and PUFAs (2). Changes in fat and carbohydrate intakes have previously been observed to alter apolipoprotein HDL concentrations. In particular higher carbohydrate intake, as is the case in the NND, has been shown to increase production rate of ApoA1 in specific HDL subfractions (30). However, increase in HDL-cholesterol and ApoA1, have also been reported for a low carbohydrate, high protein diet (31). These results are thus not in contrast with our findings, as protein intake was also higher in NND compared to ADD.

Together these results reveal that effects of changing dietary energy substrates on fatty acid synthesis and metabolism in humans are difficult to predict, and that effects of complex diets on blood lipoprotein fractions may not predict the relative health risk in a simple way but require long-term clinical trials and a larger cohort.

### Correlations of blood plasma metabolites and lipoproteins with anthropometric and clinical parameters

Acetoacetic acid occurs consistently as a marker of decreasing diastolic blood pressure (Figure 7). This ketone body has been shown to be part of a group of predictive markers for body weight loss (32). Beneficial effects have been linked to low or medium concentrations of ketone bodies, originating through fasting, exercise or ketogenic diets, whereas higher concentrations, observed for example in diabetic ketoacidosis, may be detrimental and contribute to disease morbidity (33). Higher plasma succinate levels were also found in subjects with a decrease in DBP but the result may be confounded by ketone body signals. Enhanced mitochondrial β-oxidation, which occurs for example in fasted state, can increase plasma levels of succinic acid (34) and 3-hydroxybutyrate (35). No clear metabolite pattern was found to be associated with BW losers and maintainers within the NND group, as only a few features were consistently found altered when stringent thresholds were used. The only metabolite showing a weak association with weight loss was citrate (FDR-corrected *p*-value for all citrate signals <0.1). Increasing blood plasma level of citrate was also associated with weight loss in another study (36) which linked with bone breakdown during weight loss.

## Conclusion

NMR metabolite profiling of blood plasma samples from the SHOPUS intervention study has revealed metabolite and lipoprotein pattern changes related to diet change, changes in microbial metabolites, and to body weight loss of the NND group. Increasing levels of ketone bodies were detected in the NND group and are inversely associated with the decrease in diastolic blood pressure in the NND group. Moreover, body weight changes weakly altered the metabolite and lipoprotein profile in the NND group. These results show that the unbiased and untargeted ^1^H-NMR spectroscopy enriched by the lipoprotein prediction models is able to provide a measure of the gross metabolic perturbations induced by dietary alterations and the related physiological changes experienced.

## Data availability statement

The raw data supporting the conclusions of this article will be made available by the authors, without undue reservation.

## Ethics statement

The studies involving human participants were reviewed and approved by Regional Ethics Committee of Greater Copenhagen 130 and Frederiksberg (H-3-2010-058) Danish Data Protection Agency (2007-54-0269). The patients/participants provided their written informed consent to participate in this study.

## Author contributions

LD, AA, and SE: conception and design. AT, BK, and MR: development of methodology. SE: data acquisition. AT, BK, and SE: data analysis. AT, BK, MR, LD, TL, AA, and SE: interpretation of results and writing. All authors contributed to the article and approved the submitted version.

## Funding

The study was conducted as part of the OPUS project, which is supported by a grant from the Nordea Foundation, Denmark. OPUS is an acronym of the Danish title of the project “Optimal wellbeing, development and health for Danish children through a healthy New Nordic Diet”. The trial was registered at www.clinicaltrials.gov as NCT01195610 (https://clinicaltrials.gov/ct2/show/NCT01195610). The method for calculating the lipoprotein distributions was supported by the COUNTERSTRIKE project Danish Strategic Research Council/Innovation Foundation Denmark (grant number 4105-00015B). The contribution from LD was funded by the PRIMA grant from the Novo-Nordisk Foundation (NNF19OC0056246; PRIMA—toward personalized dietary recommendations based on the interaction between diet, microbiome and abiotic conditions in the gut).

## Conflict of interest

The authors declare that the research was conducted in the absence of any commercial or financial relationships that could be construed as a potential conflict of interest.

## Publisher’s note

All claims expressed in this article are solely those of the authors and do not necessarily represent those of their affiliated organizations, or those of the publisher, the editors and the reviewers. Any product that may be evaluated in this article, or claim that may be made by its manufacturer, is not guaranteed or endorsed by the publisher.

## Supplementary material

The Supplementary material for this article can be found online at: https://www.frontiersin.org/articles/10.3389/fnut.2023.1198531/full#supplementary-material

## Figures and Tables

**Table:** 

AA	Amino Acid (and derivatives)
ADD	Average Danish Diet
ASCA	ANOVA Simultaneous Component Analysis
BW	Body Weight
AUC	Area Under the Curve
CRP	C-Reactive Protein
DBP	Diastolic Blood Pressure
DMA	Dimethylamine
FID	Free Induction Decay
FDR	False Discovery Rate
HOMA-IR	Homeostatic Model Assessment for Insulin Resistance
IDL	Intermediate Density Lipoprotein
IL6	Interleukin-6
LP	Lipoprotein
NND	New Nordic Diet
OA	Organic Acid
OH	Alcohol
PCA	Principal Component Analysis
PLS-DA	Partial Least Squares Discriminant Analysis
RG	Receiver Gain
SFA	Short Chain Fatty Acid
SS	Signature Signal
SUS	Signature signal of Unknown Spin System
SYS	Systolic Blood Pressure
TCA	Tricarboxylic Acid
TMAO	Trimethylamine-N-Oxide
TNF	Tumor Necrosis Factor

## References

[1] Scalbert A Brennan L Manach C Andres-Lacueva C Dragsted LO Draper J. The food metabolome: a window over dietary exposure. Am J Clin Nutr. (2014) 99:1286–308. doi: 10.3945/ajcn.113.076133, PMID: 24760973

[2] Poulsen SK Due A Jordy AB Kiens B Stark KD Stender S. Health effect of the new Nordic diet in adults with increased waist circumference: a 6-mo randomized controlled trial. Am J Clin Nutr. (2013) 99:35–45. doi: 10.3945/ajcn.113.069393 24257725

[3] Fritzen AM Lundsgaard A-M Jordy AB Poulsen SK Stender S Pilegaard H. New Nordic diet–induced weight loss is accompanied by changes in metabolism and AMPK signaling in adipose tissue. J Clin Endocrinol Metabol. (2015) 100:3509–19. doi: 10.1210/jc.2015-2079, PMID: 26126206

[4] Acar E Gurdeniz G Khakimov B Savorani F Korndal SK Larsen TM. Biomarkers of individual foods, and separation of diets using untargeted LC-MS-based plasma metabolomics in a randomized controlled trial. Mol Nutr Food Res. (2019) 63:e1800215. doi: 10.1002/mnfr.201800215 30094970

[5] Khakimov B Poulsen SK Savorani F Acar E Gürdeniz GZ Larsen TM. New Nordic diet versus average Danish diet: a randomized controlled trial revealed healthy long-term effects of the new Nordic diet by GC–MS blood plasma metabolomics. J Proteome Res. (2016) 15:1939–54. doi: 10.1021/acs.jproteome.6b00109, PMID: 27146725

[6] Trimigno A Khakimov B Savorani F Poulsen SK Astrup A Dragsted LO. Human urine ^1^H NMR metabolomics reveals alterations of the protein and carbohydrate metabolism when comparing habitual average Danish diet vs. healthy new Nordic diet. Nutrition. (2020) 79-80:110867. doi: 10.1016/j.nut.2020.110867 32619792

[7] Aru V Lam C Khakimov B Hoefsloot HC Zwanenburg G Lind MV. Quantification of lipoprotein profiles by nuclear magnetic resonance spectroscopy and multivariate data analysis. TrAC Trends Anal Chem. (2017) 94:210–9. doi: 10.1016/j.trac.2017.07.009

[8] Jiménez B Holmes E Heude C Tolson RF Harvey N Lodge SL. Quantitative lipoprotein subclass and low molecular weight metabolite analysis in human serum and plasma by ^1^H NMR spectroscopy in a multilaboratory trial. Anal Chem. (2018) 90:11962–71. doi: 10.1021/acs.analchem.8b02412 30211542

[9] Khakimov B Hoefsloot HCJ Mobaraki N Aru V Kristensen M Lind MV. Human blood lipoprotein predictions from ^1^H NMR spectra: protocol, model performances, and cage of covariance. Anal Chem. (2022) 94:628–36. doi: 10.1021/acs.analchem.1c01654, PMID: 34936323

[10] Savorani F Rasmussen MA Mikkelsen MS Engelsen SB. A primer to nutritional metabolomics by NMR spectroscopy and chemometrics. Food Res Int. (2013) 54:1131–45. doi: 10.1016/j.foodres.2012.12.025

[11] Otvos JD Jeyarajah EJ Bennett DW Krauss RM. Development of a proton nuclear magnetic resonance spectroscopic method for determining plasma lipoprotein concentrations and subspecies distributions from a single, rapid measurement. Clin Chem. (1992) 38:1632–8. doi: 10.1093/clinchem/38.9.1632, PMID: 1326420

[12] Khakimov B Mobaraki N Trimigno A Aru V Engelsen SB. Signature mapping (SigMa): an efficient approach for processing complex human urine ^1^H NMR metabolomics data. Anal Chim Acta. (2020) 1108:142–51. doi: 10.1016/j.aca.2020.02.025, PMID: 32222235

[13] Savorani F Tomasi G Engelsen SB. *icoshift*: a versatile tool for the rapid alignment of 1D NMR spectra. J Magn Reson. (2010) 202:190–202. doi: 10.1016/j.jmr.2009.11.012 20004603

[14] Lawton WH Sylvestre EA. Self modeling curve resolution. Technometrics. (1971) 13:617–33. doi: 10.1080/00401706.1971.10488823

[15] Monsonis Centelles S Hoefsloot HCJ Khakimov B Ebrahimi P Lind MV Kristensen M. Toward reliable lipoprotein particle predictions from NMR spectra of human blood: an interlaboratory ring test. Anal Chem. (2017) 89:8004–12. doi: 10.1021/acs.analchem.7b01329, PMID: 28692288 PMC5541326

[16] Hotelling H. Analysis of a complex of statistical variables into principal components. J Educ Psychol. (1933) 24:417–41. doi: 10.1037/h0071325

[17] Smilde AK Jansen JJ Hoefsloot HC Lamers R-JA van der Greef J Timmerman ME. ANOVA-simultaneous component analysis (ASCA): a new tool for analyzing designed metabolomics data. Bioinformatics. (2005) 21:3043–8. doi: 10.1093/bioinformatics/bti476, PMID: 15890747

[18] Ståhle L Wold S. Partial least squares analysis with cross-validation for the two-class problem: a Monte Carlo study. J Chemom. (1987) 1:185–96. doi: 10.1002/cem.1180010306

[19] Cunnane SC. Metabolism of polyunsaturated fatty acids and ketogenesis: an emerging connection. Prostaglandins Leukot Essent Fatty Acids. (2004) 70:237–41. doi: 10.1016/j.plefa.2003.11.002, PMID: 14769482

[20] Yang H Shan W Zhu F Wu J Wang Q. Ketone bodies in neurological diseases: focus on neuroprotection and underlying mechanisms. Front Neurol. (2019) 10:485. doi: 10.3389/fneur.2019.00585 31244753 PMC6581710

[21] Perry RJ Peng L Barry NA Cline GW Zhang D Cardone RL. Acetate mediates a microbiome–brain–Β-cell axis to promote metabolic syndrome. Nature. (2016) 534:213–7. doi: 10.1038/nature18309, PMID: 27279214 PMC4922538

[22] Canfora EE Blaak EE. Acetate: a diet-derived key metabolite in energy metabolism: good or bad in context of obesity and glucose homeostasis? Curr Opin Clin Nutr Metab Care. (2017) 20:477–83. doi: 10.1097/MCO.0000000000000408 28795972

[23] Kolb H Kempf K Röhling M Lenzen-Schulte M Schloot NC Martin S. Ketone bodies: from enemy to friend and guardian angel. BMC Med. (2021) 19:313. doi: 10.1186/s12916-021-02185-0, PMID: 34879839 PMC8656040

[24] Navarro SL Tarkhan A Shojaie A Randolph TW Gu H Djukovic D. Plasma metabolomics profiles suggest beneficial effects of a low-glycemic load dietary pattern on inflammation and energy metabolism. Am J Clin Nutr. (2019) 110:984–92. doi: 10.1093/ajcn/nqz169, PMID: 31432072 PMC6766441

[25] Dorokhov YL Shindyapina AV Sheshukova EV Komarova TV. Metabolic methanol: molecular pathways and physiological roles. Physiol Rev. (2015) 95:603–44. doi: 10.1152/physrev.00034.2014, PMID: 25834233

[26] Razzaghy-Azar M Nourbakhsh M Vafadar M Nourbakhsh M Talebi S Sharifi-Zarchi A. A novel metabolic disorder in the degradation pathway of endogenous methanol due to a mutation in the gene of alcohol dehydrogenase. Clin Biochem. (2021) 90:66–72. doi: 10.1016/j.clinbiochem.2021.01.007, PMID: 33539811

[27] Shindyapina AV Petrunia IV Komarova TV Sheshukova EV Kosorukov VS Kiryanov GI. Dietary methanol regulates human gene activity. PLoS One. (2014) 9:e102837. doi: 10.1371/journal.pone.0102837 25033451 PMC4102594

[28] Lanng SK Zhang Y Christensen KR Hansen AK Nielsen DS Kot W. Partial substitution of meat with insect (*Alphitobius Diaperinus*) in a carnivore diet changes the gut microbiome and metabolome of healthy rats. Foods. (2021) 10:1814. doi: 10.3390/foods10081814, PMID: 34441592 PMC8393340

[29] Ostrovsky YM. Endogenous ethanol—its metabolic, behavioral and biomedical significance. Alcohol. (1986) 3:239–47. doi: 10.1016/0741-8329(86)90032-7, PMID: 3530279

[30] Andraski AB Singh SA Lee LH Higashi H Smith N Zhang B. Effects of replacing dietary monounsaturated fat with carbohydrate on HDL (high-density lipoprotein) protein metabolism and proteome composition in humans. Arterioscler Thromb Vasc Biol. (2019) 39:2411–30. doi: 10.1161/ATVBAHA.119.312889, PMID: 31554421 PMC6874109

[31] Alzahrani AH Skytte MJ Samkani A Thomsen MN Astrup A Ritz C. Effects of a self-prepared carbohydrate-reduced high-protein diet on cardiovascular disease risk markers in patients with type 2 diabetes. Nutrients. (2021) 13:1694. doi: 10.3390/nu13051694, PMID: 34067585 PMC8157073

[32] Stroeve JH Saccenti E Bouwman J Dane A Strassburg K Vervoort J. Weight loss predictability by plasma metabolic signatures in adults with obesity and morbid obesity of the DiOGenes study. Obesity. (2016) 24:379–88. doi: 10.1002/oby.21361, PMID: 26813527

[33] Nasser S Vialichka V Biesiekierska M Balcerczyk A Pirola L. Effects of ketogenic diet and ketone bodies on the cardiovascular system: concentration matters. World J Diabetes. (2020) 11:584–95. doi: 10.4239/wjd.v11.i12.584, PMID: 33384766 PMC7754168

[34] Bjune MS Lindquist C Hallvardsdotter Stafsnes M Bjørndal B Bruheim P Aloysius TA. Plasma 3-hydroxyisobutyrate (3-HIB) and methylmalonic acid (MMA) are markers of hepatic mitochondrial fatty acid oxidation in male Wistar rats. Biochim Biophys Acta Mol Cell Biol Lipids. (2021) 1866:158887. doi: 10.1016/j.bbalip.2021.158887 33454435

[35] Mierziak J Burgberger M Wojtasik W. 3-Hydroxybutyrate as a metabolite and a signal molecule regulating processes of living organisms. Biomol Ther. (2021) 11:402. doi: 10.3390/biom11030402, PMID: 33803253 PMC8000602

[36] Papandreou C García-Gavilán J Camacho-Barcia L Toft Hansen T Harrold JA Sjödin A. Changes in circulating metabolites during weight loss are associated with adiposity improvement, and body weight and adiposity regain during weight loss maintenance: the satin study. Mol Nutr Food Res. (2021) 65:2001154. doi: 10.1002/mnfr.202001154, PMID: 34184401

