# Supplementary material for: Human blood plasma biomarkers of diet and weight loss among centrally obese subjects in a New Nordic Diet intervention
Source: Front Nutr. 2023 Jun 15;10:1198531. doi: 10.3389/fnut.2023.1198531 (PMC10308042; doi:10.3389/fnut.2023.1198531)
Supplement: Supplementary file 1 [file Data_Sheet_1.docx]

**Human blood plasma biomarkers of diet and weight loss among centrally obese subjects in a New Nordic Diet intervention**

Alessia Trimigno^1^, Bekzod Khakimov^1^, Morten Arendt Rasmussen^1+3^, Lars O. Dragsted^2^, Thomas Meinert Larsen^2^, Arne Astrup^2^, Søren Balling Engelsen^1*^

**Supporting Information**

**Table 1:** Lipoprotein dataset variables.

| **Matrix** | **Analyte** | **Name** | **Unit** |
| --- | --- | --- | --- |
| Total Plasma | Triglycerides | PlasmaTG | mg/dL |
| Main Fraction | Triglycerides | MainFracTG | mg/dL |
| Sub Fraction | Triglycerides | SubFracTG | mg/dL |
| LDL | Triglycerides | LDLTG | mg/dL |
| VLDL | Triglycerides | VLDLTG | mg/dL |
| IDL | Triglycerides | IDLTG | mg/dL |
| LDL-1 | Triglycerides | LDL-1TG | mg/dL |
| HDL-2b | Triglycerides | HDL-2bTG | mg/dL |
| HDL-2a | Triglycerides | HDL-2aTG | mg/dL |
| Total Plasma | Cholesterol | PlasmaChol | mg/dL |
| Main Fraction | Cholesterol | MainFracChol | mg/dL |
| Sub Fraction | Cholesterol | SubFracChol | mg/dL |
| LDL | Cholesterol | LDLChol | mg/dL |
| HDL | Cholesterol | HDLChol | mg/dL |
| VLDL | Cholesterol | VLDLChol | mg/dL |
| IDL | Cholesterol | IDLChol | mg/dL |
| LDL-1 | Cholesterol | LDL-1Chol | mg/dL |
| LDL-2 | Cholesterol | LDL-2Chol | mg/dL |
| LDL-3 | Cholesterol | LDL-3Chol | mg/dL |
| LDL-4 | Cholesterol | LDL-4Chol | mg/dL |
| LDL-5 | Cholesterol | LDL-5Chol | mg/dL |
| HDL-2b | Cholesterol | HDL-2bChol | mg/dL |
| HDL-2a | Cholesterol | HDL-2aChol | mg/dL |
| HDL-3 | Cholesterol | HDL-3Chol | mg/dL |
| Total Plasma | Free Cholesterol | PlasmaFreeHolest | mg/dL |
| VLDL | Free Cholesterol | VLDLFreeHolest | mg/dL |
| HDL-2b | Free Cholesterol | HDL-2bFreeHolest | mg/dL |
| Total Plasma | Phospholipids | PlasmaPhoslip | mg/dL |
| Main Fraction | Phospholipids | MainFracPhoslip | mg/dL |
| Sub Fraction | Phospholipids | SubFracPhoslip | mg/dL |
| LDL | Phospholipids | LDLPhoslip | mg/dL |
| HDL | Phospholipids | HDLPhoslip | mg/dL |
| VLDL | Phospholipids | VLDLPhoslip | mg/dL |
| LDL-1 | Phospholipids | LDL-1Phoslip | mg/dL |
| LDL-2 | Phospholipids | LDL-2Phoslip | mg/dL |
| LDL-3 | Phospholipids | LDL-3Phoslip | mg/dL |
| LDL-5 | Phospholipids | LDL-5Phoslip | mg/dL |
| HDL-2b | Phospholipids | HDL-2bPhoslip | mg/dL |
| HDL-2a | Phospholipids | HDL-2aPhoslip | mg/dL |
| Total Plasma | Apolipoprotein A1 | PlasmaApoA1 | mg/dL |
| Main Fraction | Apolipoprotein A1 | MainFracApoA1 | mg/dL |
| Sub Fraction | Apolipoprotein A1 | SubFracApoA1 | mg/dL |
| HDL | Apolipoprotein A1 | HDLApoA1 | mg/dL |
| HDL-2b | Apolipoprotein A1 | HDL-2bApoA1 | mg/dL |
| HDL-2a | Apolipoprotein A1 | HDL-2aApoA1 | mg/dL |
| HDL-3 | Apolipoprotein A1 | HDL-3ApoA1 | mg/dL |
| Total Plasma | Apolipoprotein B | PlasmaApoB | mg/dL |
| Main Fraction | Apolipoprotein B | MainFracApoB | mg/dL |
| Sub Fraction | Apolipoprotein B | SubFracApoB | mg/dL |
| LDL | Apolipoprotein B | LDLApoB | mg/dL |
| VLDL | Apolipoprotein B | VLDLApoB | mg/dL |
| IDL | Apolipoprotein B | IDLApoB | mg/dL |
| LDL-1 | Apolipoprotein B | LDL-1ApoB | mg/dL |
| LDL-2 | Apolipoprotein B | LDL-2ApoB | mg/dL |
| LDL-3 | Apolipoprotein B | LDL-3ApoB | mg/dL |
| LDL-4 | Apolipoprotein B | LDL-4ApoB | mg/dL |
| LDL-5 | Apolipoprotein B | LDL-5ApoB | mg/dL |
| LDL-6 | Apolipoprotein B | LDL-6ApoB | mg/dL |
| Total Plasma | Cholesterol Esters | PlasmaCE | mg/dL |
| VLDL | Cholesterol Esters | VLDLCE | mg/dL |
| IDL | Cholesterol Esters | IDLCE | mg/dL |
| LDL | Cholesterol Esters | LDLCE | mg/dL |
| LDL-1 | Cholesterol Esters | LDL-1CE | mg/dL |
| LDL-3 | Cholesterol Esters | LDL-3CE | mg/dL |
| HDL-2b | Cholesterol Esters | HDL-2bCE | mg/dL |

**Supplementary Table 2:** Anthropometric and clinical parameters variables.

| Age (y) |
| --- |
| Body weight (kg) |
| BMI (kg/m2) |
| Systolic Blood Pressure (mm Hg) |
| Diastolic Blood Pressure (mm Hg) |
| Triglycerides (mmol/L) |
| Total Cholesterol (mmol/L) |
| HDL Cholesterol (mmol/L) |
| LDL Cholesterol (mmol/L) |
| VLDL Cholesterol (mmol/L) |
| Plasma Glucose at time 0 min (mmol/L) |
| Plasma Glucose at time 120 min (mmol/L) ^∫^ |
| Serum Insulin at time 0 min (pmol/L) |
| Serum Insulin at time 120 min (pmol/L) ^∫^ |
| HOMA-IR* |
| Matsuda index ^∫^ |
| Matsuda based on weighted average ^∫^ |
| Plasma TNF*-alpha (pg/mL) |
| Plasma IL*-6 (pg/mL) |
| Plasma CRP* (mg/L) |

* HOMA-IR was calculated as follows: fasting plasma glucose (mmol/L) × fasting plasma insulin (μU/mL) ÷ 22.5;

^∫^ The Matsuda index was calculated as follows: 10,000 ÷ √{[fasting glucose (mg/dL) × fasting insulin (μU/mL)] × [mean glucose (mg/dL) × mean insulin (μU/mL) concentrations during oral-glucose-tolerance test)]}; TNF: Tumor Necrosis Factor; IL-6: Interleukin 6; CRP= C-reactive protein; ^∫^variables were collected only at T0 and T2.

**Supplementary Table 3.** Dietary and nutritional data. All variables were then divided by bodyweight at T1 and T2, respectively.

| Time-point | Variable |
| --- | --- |
| T1 and T2 (calculated on food diaries) | Mass (g) |
|  | Energy (kJ/day) |
|  | Density (kJ/100g) |
|  | Protein (E%) |
|  | Carbs (E %) |
|  | Fiber (g/10MJ) |
|  | Sugar (E %) |
|  | Fat (E %) |
|  | SFA (E %) |
|  | MUFA (E %) |
|  | PUFA (E %) |
|  | Alcohol (E %) |
|  | Protein (g) |
|  | Fat (g) |
|  | Carb (g) |
|  | Sugar (g) |
|  | Alcohol (g) |
|  | Fiber (g) |
|  | SFA (g) |
|  | MUFA (g) |
|  | PUFA (g) |
| T2 (calculated on shop purchases) | Shop days |
|  | Energy (kJ) |
|  | Protein (E%) |
|  | Fat (E %) |
|  | SFA (E %) |
|  | MUFA (E %) |
|  | PUFA (E %) |
|  | Carbs (E %) |
|  | Sugar (E %) |
|  | Alcohol (E %) |
|  | Fiber (g) |
|  | Organic food (% of total amount of food) |
|  | Nordic food (% of total amount of food) |
|  | Fruit (g/10MJ) |
|  | Vegetables (g/10MJ) |
|  | Berries (g/10MJ) |
|  | Cabbages (g/10MJ) |
|  | Root vegetables (g/10MJ) |
|  | Legumes (g/10MJ) |
|  | Wholegrain (g/10MJ) |
|  | Meat (g/10MJ) |
|  | Nuts (g/10MJ) |
|  | Potatoes (g/10MJ) |
|  | Spices (g/10MJ) |
|  | Wild plants and mushrooms (g/10MJ) |
|  | Seaweeds (g/10MJ) |
|  | Fish (g/10MJ) |
|  | Game meat (g/10MJ) |
|  | Milk (g/10MJ) |
|  | density (kJ/100g) |
|  | mass (g) |
|  | Energy (kJ/day) |
|  | Protein (g) |
|  | Fat (g) |
|  | SFA (g) |
|  | MUFA (g) |
|  | PUFA (g) |
|  | Carbs (g) |
|  | Sugar (g) |
|  | Alcohol (g) |
|  | Fiber (g) |
|  | Organic food (g) |
|  | Nordic food (g) |
|  | Fruit (g) |
|  | Vegetables (g) |
|  | Berries (g) |
|  | Cabbages (g) |
|  | Root vegetables (g) |
|  | Legumes (g) |
|  | Wholegrain (g) |
|  | Meat (g) |
|  | Nuts (g) |
|  | Potatoes (g) |
|  | Spices (g) |
|  | Wild plants and mushrooms (g) |
|  | Seaweeds (g) |
|  | Fish (g) |
|  | Game meat (g) |
|  | Milk (g) |

**Supplementary Table 4.** An overview of datasets and data analysis methods applied in this study.

| Investigated Factor | Dataset | Size | Note |
| --- | --- | --- | --- |
| D | **ΔX**_T1_CPMG_ | 146x154 | CPMG NMR data at T1 after baseline (T0) correction |
| D | **ΔX**_T2_CPMG_ | 146x154 | CPMG NMR data at T2 after baseline (T0) correction |
| D | **ΔX**_T1_NOESY_ | 146x154 | NOESY NMR data at T1 after baseline (T0) correction |
| D | **ΔX**_T2_NOESY_ | 146x154 | NOESY NMR data at T2 after baseline (T0) correction |
| D, N | **ΔX**_T1_LP_ | 146x105 | Lipoprotein NMR data at T1 after baseline (T0) correction |
| D, N | **ΔX**_T2_LP_ | 146x105 | Lipoprotein NMR data at T2 after baseline (T0) correction |
| C | **X**_T1_META_ | 146x16 | Anthropometric and clinical parameters variables at T1 |
| C | **X**_T2_META_ | 146x20 | Anthropometric and clinical parameters variables at T2 |
| N | **X**_T1_DIET_ | 146x21 | Dietary and nutritional variables at T1 (from food diaries) |
| N | **X**_T2_DIET_ | 146x80 | Dietary and nutritional variables at T2 (from food diaries and shop data) |
| B | **X**_T0_NND_BW_ | 41x413 | CPMG, NOESY and Lipoprotein NMR data at T0 for NND subjects with the highest/lowest BW loss both at T1 and T2. |
| B | **X**_T1_NOESY_NND_BW_ | 52x154 | NOESY NMR data at T1 for NND subjects with the highest/lowest BW loss both at T1. |
| B | **X**_T2_NOESY_NND_BW_ | 62x154 | NOESY NMR data at T2 for NND subjects with0 the highest/lowest BW loss both at T2. |
| B | **X**_T1_CPMG_NND_BW_ | 52x154 | CPMG NMR data at T1 for NND subjects with the highest/lowest BW loss both at T1. |
| B | **X**_T2_CPMG_NND_BW_ | 62x154 | CPMG NMR data at T2 for NND subjects with the highest/lowest BW loss both at T2. |
| B | **X**_T1_LP_NND_BW_ | 52x105 | Lipoprotein NMR data at T1 for NND subjects with the highest/lowest BW loss both at T1. |
| B | **X**_T2_LP_NND_BW_ | 62x105 | Lipoprotein NMR data at T2 for NND subjects with the highest/lowest BW loss both at T2. |

* D = diet effect, B = body weight loss, C = correlation with anthropometric and clinical data, N= correlation with nutritional/dietary data.

**Supplementary Table 5.** List of variables included in the CPMG and NOESY datasets after interval selection through SigMa and corresponding mean chemical shift in ppm. For known signals, the corresponding protons, multiplicity and J constant are reported.

| **Interval name** | **Mean chemical shift (ppm)** |
| --- | --- |
| 2-aminobutyric acid (**C*H_3_***, t, J = 7.0 Hz) | 1.01 |
| 3-hydroxybutyric acid (**C*H_3_***, d, J = 6.26 Hz) | 1.23 |
| acetic acid (**C*H_3_***, s) | 1.94 |
| acetoacetic acid (**C*H_2_***, s) | 3.47 |
| acetone (**C*H_3_***, s) | 2.26 |
| alanine (**C*H_3,_*** d, J = 7.14 Hz) | 1.51 |
| aspartic acid1 (**C*H_2,_*** dd, J = 6.4 Hz) | 2.69 |
| betaine1 (**C*H_3,_*** s) | 3.28 |
| CaEDTA2 | 2.59 |
| cis-aconitic acid2 (**C*H_,_*** s) | 5.77 |
| citric acid1 (**C*H_2,_*** d, J = 2 Hz) | 2.56 |
| citric acid2 (**C*H_2,_*** d, J = 1.95 Hz) | 2.67 |
| citric acid3 (**C*H_2,_*** d, J = 1.95 Hz, part of doublet with citric acid2) | 2.70 |
| creatinine_creatine_creatinephosphate (**C*H_3_***, s) | 3.07 |
| creatine2 (**C*H_2_***, s) | 3.96 |
| creatinine2 (**C*H_2_***, s) | 4.08 |
| dimethylamine (**C*H_3_***, s) + MgEDTA | 2.73 |
| dimethylglycine1 (**C*H_3_***, s) | 2.95 |
| dimethylsulfone (**C*H_3_***, s) | 3.18 |
| ethanol (**C*H_3,_*** t, J = 7.08 Hz) | 1.20 |
| ethanol2 (**C*H_2,_*** q, J = 7.07 Hz) | 3.69 |
| freeEDTA | 3.64 |
| formic acid (**C*H***, s) | 8.49 |
| fumaric acid (**C*H_2_***, s) | 6.61 |
| galactose (**O*H***, d, J = 3.66 Hz) | 5.26 |
| glucose1 (**C*H***, dd, J = 8.4, 6.2 Hz) | 3.45 |
| glucose2 (**C*H***, dd, J = 8.4, 6.2 Hz) | 3.48 |
| glucose3 (**C*H***, dd, J = 1.7, 1.6 Hz) | 3.52 |
| glucose4 (**C*H***, dd, J = 1.7, 1.6 Hz) | 3.58 |
| glucose5 (**C*H_2,_*** d, J = 5.4 Hz) | 3.76 |
| glucose6 (**C*H***, dt, J = 8.4, 5.4 Hz) | 3.81 |
| glucose7 (**C*H***, dt, J = 8.4, 5.4 Hz) _sucrose (**C*H***, dd, J = 3.25, 6.46 Hz) | 3.86 |
| glucose8 (**C*H***, dd, J = 6.2, 1.7 Hz) | 3.92 |
| glucose9 (**O*H***, d, J = 1.6 Hz) | 5.27 |
| glutamine1 (**C*H_2_***, m, J = 6.85, 5.75, 2.94 Hz)_methionine (**C*H_3_***, m) | 2.17 |
| glutamine2 (**C*H_2_***, m, J = 7.80, 7.66 Hz) | 2.48 |
| glutamine3 (**C*H***, t, J = 6.16 Hz) | 3.78 |
| glycine (**C*H_2_***, s) | 3.59 |
| isobutyric acid (**C*H_3_***, d, J = 7.02 Hz) | 1.11 |
| isoleucine1 (**C*H_3_***, t, J = 7.414 Hz) | 0.97 |
| isoleucine2 (**C*H_3_***, d, J = 7.001 Hz) | 1.03 |
| lactic acid1 (**C*H_3_***, d, J = 6.96 Hz)_threonine (**C*H_3_***, d, J = 6.585 Hz) | 1.36 |
| lactic acid2 (**C*H***, q, J = 6.93 Hz) | 4.14 |
| leucine (**C*H_3_***, t, J = 5.897 Hz) | 0.99 |
| lysine1 (**C*H_2_***, tt, J = 6.6, 6.5 Hz) | 1.74 |
| lysine2 (**C*H_2_***, dtd, J = 14.4, 6.6, 6.2 Hz) | 1.92 |
| lysine3 (**C*H_2_***, t, J = 6.5 Hz) | 3.06 |
| methanol (**C*H_3_***, s) | 3.39 |
| methine_LP | 5.33 |
| methionine2 (**C*H_2_***, t, J = 7.587 Hz)_aspartate4 (**C*H_2_***, dd) | 2.66 |
| methyl_LP | 0.89 |
| Methylen_LP | 1.30 |
| MgEDTA2 | 3.24 |
| phenylalanine1 (**C*H***, m) | 7.37 |
| phenylalanine2 (**C*H***, m) | 7.46 |
| pyruvic acid (**C*H_3_***, s) | 2.40 |
| sarcosine1 (**C*H_3_***, s) | 2.74 |
| serine1 (**C*H***, dd, J = 5.583, 3.80 Hz) | 3.97 |
| serine2 (**C*H_2_***, m)_asparagine2(**C*H***, dd, J = 7.69, 4.26 Hz) | 4.00 |
| succinic acid (**C*H_2_***, s) | 2.43 |
| TMAO (**C*H_3_***, s) | 3.26 |
| tyrosine1 (**C*H_2_***, m, J = 1.575 Hz) | 6.93 |
| tyrosine2 (**C*H_2_***, m, J = 1.604 Hz) | 7.22 |
| valine1 (**C*H_3_***, d, J = 7.01 Hz) | 1.01 |
| valine2 (**C*H_3_***, d, J = 7.05 Hz) | 1.06 |
| SUS1_s | 0.72 |
| SUS2_d | 1.15 |
| SUS3_d | 1.44 |
| SUS4_s | 1.60 |
| SUS5_s | 2.07 |
| SUS6_dd | 2.29 |
| SUS7_s | 2.31 |
| SUS8_d | 2.33 |
| SUS9_d | 2.34 |
| SUS10_s | 2.36 |
| SUS11_d | 2.37 |
| SUS12_d | 2.38 |
| SUS13_s | 2.40 |
| SUS14_d | 2.42 |
| SUS15_s | 2.44 |
| SUS16_s | 2.45 |
| SUS17_s | 2.89 |
| SUS18_d_or_ss | 3.10 |
| SUS19_s | 3.11 |
| SUS20_d | 3.14 |
| SUS21_s | 3.22 |
| SUS22_s | 3.29 |
| SUS23_s | 3.30 |
| SUS24_s | 3.31 |
| SUS25_s | 3.32 |
| SUS26_s | 3.35 |
| SUS27_s_and ethanol (**C*H_2_***, q, J = 7.07 Hz) | 3.67 |
| SUS28_d | 5.94 |
| SUS29_s | 7.08 |
| SUS30_m | 7.68 |
| SUS31_s | 7.70 |
| SUS32_s | 7.87 |
| SUS33_s | 7.89 |
| bin1 | 1.90 |
| bin2 | 2.03 |
| bin3 | 2.11 |
| bin4 | 2.25 |
| bin5 | 2.45 |
| bin6_aspartate_asparagine | 2.77 |
| bin7 | 2.93 |
| bin8 | 2.98 |
| bin9 | 3.04 |
| bin10_with_s | 3.19 |
| bin11 | 3.35 |
| bin12 | 3.38 |
| bin13 | 3.41 |
| bin14_threonine_sarcosine_valine | 3.61 |
| bin15 | 3.90 |
| bin16 | 4.10 |
| bin17 | 4.16 |
| bin18 | 4.29 |
| bin19 | 5.21 |
| bin20 | 6.14 |
| bin21 | 6.84 |
| bin22 | 6.98 |
| bin23 | 7.06 |
| bin24 | 7.11 |
| bin25 | 7.20 |
| bin26 | 7.28 |
| bin27 | 7.40 |
| bin28 | 7.49 |
| bin29 | 7.80 |
| bin30 | 8.21 |
| bin31 | 0.50 |
| bin32 | 0.82 |
| bin33 | 0.92 |
| bin34 | 1.12 |
| bin35 | 1.16 |
| bin36 | 1.37 |
| bin37 | 1.49 |
| bin38 | 1.56 |
| bin39 | 1.70 |
| bin40 | 2.20 |
| bin41 | 2.28 |
| bin42 | 2.51 |
| bin43 | 2.58 |
| bin44 | 2.60 |
| bin45 | 3.35 |
| bin46 | 3.72 |
| bin47 | 3.83 |
| bin48 | 4.07 |
| bin49 | 4.21 |
| bin50 | 4.38 |
| bin51 | 5.40 |
| bin52 | 5.87 |
| bin53 | 7.47 |
| bin54 | 7.63 |
| bin55 | 7.92 |
| bin56 | 8.52 |

**Supplementary Table 6:** Results from ASCA on diet for each of the different matrices (CPMG, NOESY and LP dataset) at T1, and T2.

|  | **DIET** | |
| --- | --- | --- |
| Matrix | % variation | p-value |
| **ΔX**_T1_CPMG_ | 1.8 | 2.3∙10^-3^ |
| **ΔX**_T2_CPMG_ | 3.6 | 2.7∙10^-6^ |
| **ΔX**_T1_NOESY_ | 2.0 | 6.1∙10^-3^ |
| **ΔX**_T2_NOESY_ | 4.8 | 5.1∙10^-6^ |
| **ΔX**_T1_LP_ | 0.4 | 0.691 |
| **ΔX**_T2_LP_ | 3.2 | 4.1∙10^-3^ |

**Supplementary Table 7:** A list of discriminative variables (SUS and bins, ordered numerically for each matrix) for the diet effect identified by PLS-DA variable selection and one-way ANOVA with FDR-correction. Median variance is calculated as the median of the individual % variance from T0.

| **Variable name** | **p-value** | **Effect size** | **Median NND % variation** | **Median ADD % variation** | **Matrix** |
| --- | --- | --- | --- | --- | --- |
| SUS22_s | 3.29E-04 | 13.70 | 7.46 | -4.49 | ΔX_T1_NOESY_ |
| SUS25_s | 2.12E-05 | 17.59 | -1.04 | 1.86 | ΔX_T1_NOESY_ |
| SUS1_s | 7.18E-04 | 10.00 | 0.43 | -0.60 | ΔX_T2_NOESY_ |
| SUS5_s | 9.48E-03 | 5.70 | 4.39 | -4.38 | ΔX_T2_NOESY_ |
| SUS6_dd | 4.93E-02 | 3.43 | 6.51 | -2.74 | ΔX_T2_NOESY_ |
| SUS8_d | 7.18E-04 | 9.98 | 1.95 | -2.32 | ΔX_T2_NOESY_ |
| SUS9_d | 6.18E-04 | 10.40 | 2.63 | -3.34 | ΔX_T2_NOESY_ |
| SUS12_d | 4.27E-02 | 3.64 | 1.00 | -0.48 | ΔX_T2_NOESY_ |
| SUS14_d | 1.78E-04 | 12.55 | 1.92 | -4.98 | ΔX_T2_NOESY_ |
| SUS17_s | 6.72E-03 | 6.29 | 6.12 | -4.52 | ΔX_T2_NOESY_ |
| SUS19_s | 2.69E-04 | 11.75 | 4.35 | -4.52 | ΔX_T2_NOESY_ |
| SUS20_d | 2.12E-04 | 12.23 | 5.16 | -4.07 | ΔX_T2_NOESY_ |
| SUS22_s | 9.88E-05 | 13.64 | 4.75 | -3.97 | ΔX_T2_NOESY_ |
| SUS25_s | 1.70E-02 | 4.88 | -0.08 | 0.58 | ΔX_T2_NOESY_ |
| SUS28_b | 4.53E-03 | 6.86 | 5.57 | -4.10 | ΔX_T2_NOESY_ |
| SUS22_s | 3.32E-04 | 13.69 | 7.04 | -5.83 | ΔX_T1_CPMG_ |
| SUS25_s | 1.71E-06 | 20.34 | -0.96 | 2.03 | ΔX_T1_CPMG_ |
| SUS3_d | 6.60E-03 | 6.89 | 0.59 | -0.58 | ΔX_T2_CPMG_ |
| SUS5_s | 9.45E-03 | 6.22 | 4.21 | -3.07 | ΔX_T2_CPMG_ |
| SUS6_dd | 3.69E-03 | 8.36 | 10.14 | -4.28 | ΔX_T2_CPMG_ |
| SUS7_s | 1.67E-02 | 5.30 | 3.33 | -1.98 | ΔX_T2_CPMG_ |
| SUS8_d | 5.32E-04 | 11.86 | 1.57 | -2.96 | ΔX_T2_CPMG_ |
| SUS9_d | 5.83E-04 | 11.42 | 1.76 | -4.25 | ΔX_T2_CPMG_ |
| SUS11_d | 4.74E-02 | 3.81 | 0.83 | -0.78 | ΔX_T2_CPMG_ |
| SUS12_d | 1.93E-02 | 5.10 | 0.96 | -0.92 | ΔX_T2_CPMG_ |
| SUS14_d | 5.32E-04 | 11.93 | 1.90 | -4.46 | ΔX_T2_CPMG_ |
| SUS17_s | 2.45E-02 | 4.72 | 4.40 | -2.14 | ΔX_T2_CPMG_ |
| SUS19_s | 6.10E-03 | 7.08 | 6.62 | -4.02 | ΔX_T2_CPMG_ |
| SUS20_d | 5.32E-04 | 12.15 | 4.94 | -3.94 | ΔX_T2_CPMG_ |
| SUS22_s | 8.69E-04 | 10.63 | 4.84 | -3.20 | ΔX_T2_CPMG_ |
| SUS25_s | 8.02E-03 | 6.45 | 0.12 | 1.67 | ΔX_T2_CPMG_ |
| bin1 1.90 ppm | 1.83E-03 | 8.58 | 5.81 | -4.63 | ΔX_T2_NOESY_ |
| bin3 2.11 ppm | 3.70E-03 | 7.15 | 6.04 | -4.03 | ΔX_T2_NOESY_ |
| bin6_aspartate_asparagine 2.77 ppm | 3.01E-02 | 4.12 | 0.16 | -0.24 | ΔX_T2_NOESY_ |
| bin8 2.98 ppm | 2.19E-05 | 15.54 | 5.46 | -5.85 | ΔX_T2_NOESY_ |
| bin9 3.04 ppm | 3.88E-04 | 11.08 | 4.22 | -7.35 | ΔX_T2_NOESY_ |
| bin10_with_s 3.19 ppm | 1.94E-03 | 8.43 | 7.69 | -2.74 | ΔX_T2_NOESY_ |
| bin17 4.16 ppm | 2.51E-03 | 7.78 | 0.39 | -1.15 | ΔX_T2_NOESY_ |
| bin20 6.14 ppm | 5.58E-06 | 17.53 | 5.75 | -7.06 | ΔX_T2_NOESY_ |
| bin21 6.84 ppm | 1.95E-03 | 8.39 | 6.06 | -5.44 | ΔX_T2_NOESY_ |
| bin22 6.98 ppm | 7.80E-03 | 6.02 | 8.72 | -5.31 | ΔX_T2_NOESY_ |
| bin23 7.06 ppm | 7.80E-03 | 6.02 | 7.21 | -6.90 | ΔX_T2_NOESY_ |
| bin24 7.11 ppm | 1.93E-02 | 4.69 | 8.81 | -4.51 | ΔX_T2_NOESY_ |
| bin26 7.33 ppm | 1.00E-06 | 19.73 | 7.47 | -9.10 | ΔX_T2_NOESY_ |
| bin27 7.40 ppm | 7.39E-03 | 6.13 | 0.55 | -0.88 | ΔX_T2_NOESY_ |
| bin28 7.49 ppm | 1.89E-05 | 15.91 | 6.19 | -6.90 | ΔX_T2_NOESY_ |
| bin29 7.80 ppm histidine | 1.78E-04 | 12.57 | 6.09 | -6.23 | ΔX_T2_NOESY_ |
| bin30 8.21 ppm | 4.48E-04 | 10.84 | 5.00 | -6.09 | ΔX_T2_NOESY_ |
| bin32 0.82 ppm | 2.21E-03 | 8.16 | 4.95 | -3.60 | ΔX_T2_NOESY_ |
| bin34 1.12 ppm | 3.91E-02 | 3.77 | 0.15 | -0.65 | ΔX_T2_NOESY_ |
| bin39 1.70 ppm | 1.32E-02 | 5.21 | 7.20 | -4.43 | ΔX_T2_NOESY_ |
| bin40 2.20 ppm | 2.23E-03 | 8.12 | 0.47 | -0.68 | ΔX_T2_NOESY_ |
| bin42 2.51 ppm | 5.41E-03 | 6.61 | 12.18 | -7.73 | ΔX_T2_NOESY_ |
| bin43 2.58 ppm | 1.75E-03 | 8.72 | 0.68 | -0.54 | ΔX_T2_NOESY_ |
| bin44 2.6 ppm | 2.24E-03 | 8.08 | 6.34 | -3.83 | ΔX_T2_NOESY_ |
| bin50 4.38 ppm | 4.86E-02 | 3.46 | 0.16 | -0.37 | ΔX_T2_NOESY_ |
| bin52 5.4 ppm | 1.19E-04 | 13.27 | 5.00 | -4.73 | ΔX_T2_NOESY_ |
| bin8 2.98 ppm | 1.40E-02 | 8.82 | 3.99 | -5.62 | ΔX_T1_CPMG_ |
| bin1 1.90 ppm | 1.05E-02 | 6.04 | 3.26 | -4.21 | ΔX_T2_CPMG_ |
| bin3 2.11 ppm | 3.69E-03 | 8.32 | 5.68 | -3.97 | ΔX_T2_CPMG_ |
| bin8 2.98 ppm | 2.93E-04 | 13.36 | 6.49 | -7.39 | ΔX_T2_CPMG_ |
| bin9 3.04 ppm | 2.58E-02 | 4.64 | 1.24 | -1.77 | ΔX_T2_CPMG_ |
| bin10_with_s 3.19 ppm | 4.99E-03 | 7.43 | 7.58 | -1.45 | ΔX_T2_CPMG_ |
| bin17 4.16 ppm | 8.69E-04 | 10.57 | 0.23 | -1.73 | ΔX_T2_CPMG_ |
| bin29 7.80 ppm histidine | 2.45E-02 | 4.72 | 3.24 | -2.71 | ΔX_T2_CPMG_ |
| bin32 0.82 ppm | 4.92E-03 | 7.53 | 3.89 | 10.60 | ΔX_T2_CPMG_ |
| bin38 1.56 | 5.22E-03 | 7.33 | 8.77 | -3.67 | ΔX_T2_CPMG_ |
| bin43 2.58 ppm | 4.92E-03 | 7.49 | 0.48 | -0.37 | ΔX_T2_CPMG_ |
| bin44 2.6 ppm | 4.68E-02 | 3.85 | 7.74 | -4.40 | ΔX_T2_CPMG_ |
| bin49 4.21 ppm | 1.27E-02 | 5.72 | 3.65 | -4.69 | ΔX_T2_CPMG_ |
| bin52 5.4 ppm | 4.57E-02 | 3.90 | -0.06 | -0.08 | ΔX_T2_CPMG_ |

**Supplementary Table 8.** LP features recurrently significant in PLS variable selection and ANOVA without FDR-correction.

| **Matrix** | **Feature** |
| --- | --- |
| ΔX_T1_LP_NND_BW_ | Main Fraction TG |
| ΔX_T1_LP_NND_BW_ | Subfraction TG |
| ΔX_T1_LP_NND_BW_ | Plasma Cholesterol |
| ΔX_T1_LP_NND_BW_ | Main Fraction Cholesterol |
| ΔX_T1_LP_NND_BW_ | Plasma Free Cholesterol |
| ΔX_T1_LP_NND_BW_ | LDL-5 Phospholipids |
| ΔX_T1_LP_NND_BW_ | Plasma Apolipoprotein B |
| ΔX_T1_LP_NND_BW_ | LDL-5 Phospholipids |
| ΔX_T2_LP_NND_BW_ | LDL TG |
| ΔX_T2_LP_NND_BW_ | IDL TG |
| ΔX_T2_LP_NND_BW_ | LDL-1 TG |
| ΔX_T2_LP_NND_BW_ | HDL-2b TG |
| ΔX_T2_LP_NND_BW_ | LDL-1 Cholesterol |
| ΔX_T2_LP_NND_BW_ | LDL-2 Cholesterol |
| ΔX_T2_LP_NND_BW_ | LDL-1 Apolipoprotein B |
| ΔX_T2_LP_NND_BW_ | LDL-2 Apolipoprotein B |
| ΔX_T2_LP_NND_BW_ | LDL-1 CE |

**Supplementary Table 9.** ASCA based decomposition of variance according to the delta anthropometric and clinical parameters factors, considering the two extreme tertiles of the population for each delta anthropometric and clinical parameter. Only matrices reporting significant impact of the anthropometric and clinical parameters on the metabolome are reported.

| **Matrix** | **Factor** | **Variance (%)** | **p-value** | **Number of significant features** |
| --- | --- | --- | --- | --- |
| **ΔX**_T1_NOESY_NND_ | ΔDBP | 3.9 | 0.034 | 6 |
| **ΔX**_T1_CPMG_NND_ | ΔDBP | 4.1 | 0.011 | 11 |
| **ΔX**_T2_CPMG_NND_ | ΔDBP | 3.4 | 0.013 | 9 |

**Supplementary Table 10.** Significant metabolite in relation to diastolic blood pressure (DBP) changes in the different spectral matrices, with reported effect size and p-value, and comparison between high/low ΔDBP class. ↓ = lower value in high ΔDBP class, ↑ = higher value in high ΔDBP class.

| **ΔX_T1_NOESY_NND_ *DBP*** | ***Effect Size (%)*** | ***p-value*** | ***Comparison high ΔDBP/low ΔDBP*** | **ΔX_T1_CPMG_NND_ *DBP*** | ***Effect Size (%)*** | ***p-value*** | ***Comparison high ΔDBP/low ΔDBP*** | **ΔX_T2_CPMG_NND_ *DBP*** | ***Effect Size (%)*** | ***p-value*** | ***Comparison high ΔDBP/low ΔDBP*** |
| --- | --- | --- | --- | --- | --- | --- | --- | --- | --- | --- | --- |
| Acetoacetic acid | 22.67 | 3.25E-04 | ↓ (-1.6% in high DBP, +4.5% in low DBP) | acetone | 17.71 | 1.95E-03 | ↓ (+3.8% in high DBP, +52.6% in low DBP) | 3-hydroxybutyric acid | 20.19 | 6.49E-04 | ↓ (-1.1 % in high DBP, +3.2% in low DBP) |
|  |  |  |  | Succinic acid | 15.83 | 2.92E-03 | ↓ (-0.9% in high DBP, +3.5% in low DBP) | Acetoacetic acid | 19.4 | 1.30E-03 | ↓ (-1.4% in high DBP, +4.5% in low DBP) |
|  |  |  |  |  |  |  |  | acetone | 16.78 | 1.95E-03 | ↓ (-2.4% in high DBP, +30.7% in low DBP) |
|  |  |  |  |  |  |  |  | Succinic acid | 15.54 | 2.60E-03 | ↓ (-2.0% in high DBP, +3.5% in low DBP) |


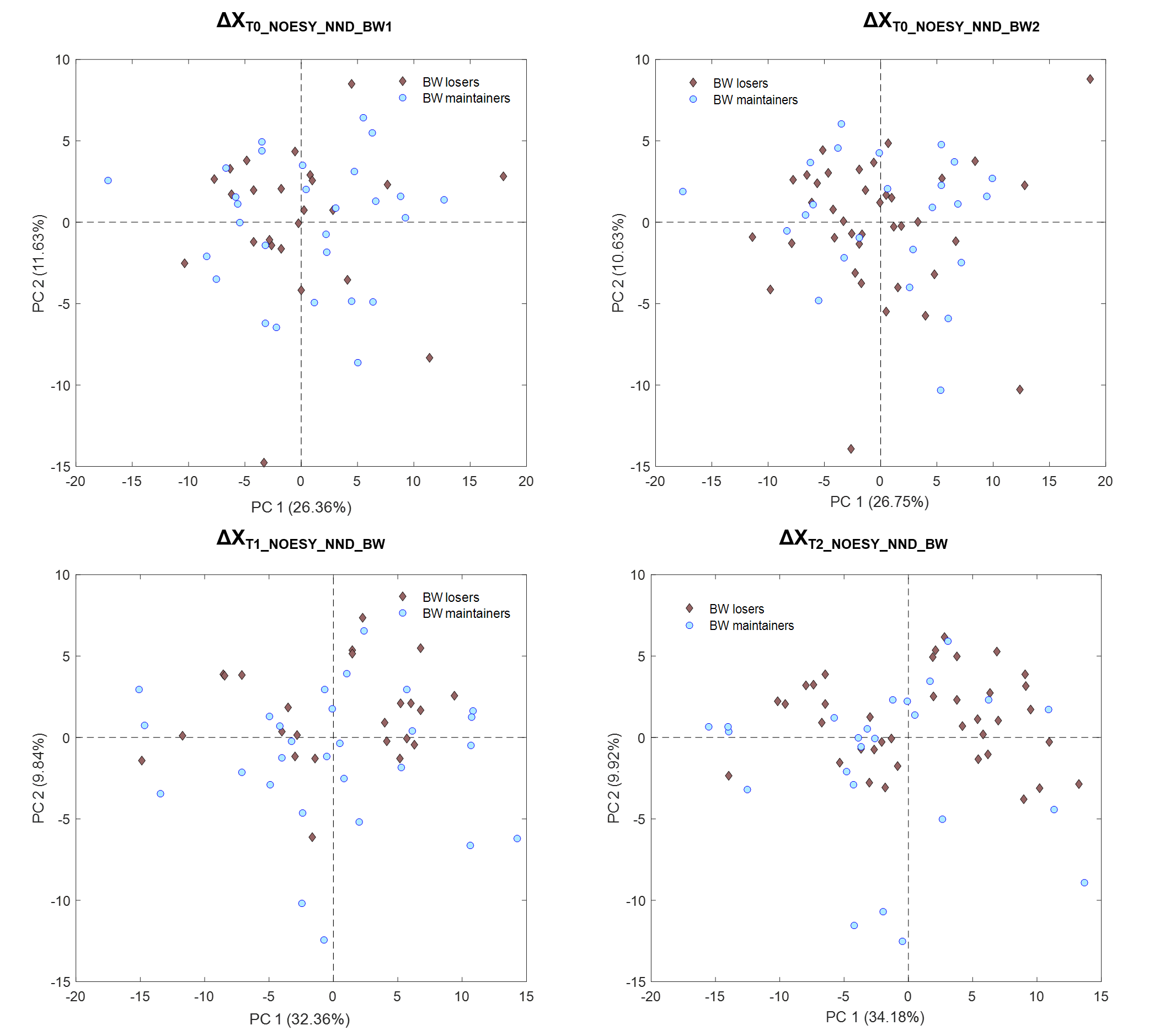
 **Supplementary Figure 1.** PCA scoreplots for PC1 and PC2 showing lack of separation between BW losers and maintainers in the different NOESY matrices. For the XT0_NOESY_NND_BW dataset subjects considered BW losers and maintainers at T1 or at T2 were selected, respectively, for the top left and top right figures.
